# Comparative assessment of food-grade osmolytes for enhancing yeast fermentation performance under salt stress

**DOI:** 10.1128/spectrum.00102-26

**Published:** 2026-03-30

**Authors:** Gunaseelan Sathaiah, Pin-Cheng Chen, Myat Min Khant, Prasanth K. S. Pillai

**Affiliations:** 1 Department of Food Science and Nutrition, University of Minnesota 311837 , St. Paul, Minnesota, USA; University of Mississippi, University, Mississippi, USA

**Keywords:** osmolyte, osmoprotectant, osmotic stress, *Saccharomyces cerevisiae*, high salt stress

## Abstract

High salt concentrations impose severe osmotic and oxidative stress on *Saccharomyces cerevisiae*, limiting growth and fermentation efficiency in food industrial processes. Osmolytes, small compatible solutes such as sugars, polyols, and amino acids, can protect cells by stabilizing proteins, preserving water balance, and maintaining cellular integrity. While individual osmolytes have been studied, no systematic assessment across diverse commercial classes under uniform stress conditions has been reported. Here, we evaluated 12 commercially relevant osmolytes representing sugars (trehalose, sucrose, and lactose), sugar alcohols and polyols (sorbitol, mannitol, xylitol, *myo-*inositol, and glycerol), amino acids (proline, glycine, and β-alanine), and urea for their ability to mitigate 6% sodium chloride (NaCl) stress in yeast. Growth performance was quantified using optical density, viability, budding efficiency, cell morphology, biomass productivity, and multivariate analysis. Osmolyte supplementation strongly modulated yeast stress responses. *Myo-*inositol, sucrose, and lactose emerged as the most effective treatments, restoring growth up to ~85% of unstressed controls and increasing viability by >70% compared to salt-stressed cells. These osmolytes enhanced antioxidant capacity (up to +47%) and restored budding frequency and cell shrinkage, indicating partial recovery of normal morphogenesis. Principal component analysis and heatmap clustering distinguished osmolyte classes, with sucrose, lactose, and *myo-*inositol clustering with unstressed controls, underscoring their superior osmoprotective potential. This study provides the first direct comparative ranking of commercial osmolytes, identifying *myo-*inositol, sucrose, and lactose as the superior osmoprotectants for mitigating salt-induced stress in *S. cerevisiae*. These osmolytes enhance growth, redox balance, and morphogenesis, providing a quantitative framework for selecting osmoprotectants to improve productivity in high-osmolarity industrial fermentations or high-gravity fermentation.

**IMPORTANCE:**

*Saccharomyces cerevisiae* plays a vital role in brewing, baking, alternative protein production, and food biotechnology, but its performance often declines under high salt conditions typical of industrial fermentations. Salt stress causes water loss, cell shrinkage, and oxidative damage, resulting in impaired metabolism and reduced productivity. This limitation is increasingly significant as sustainable bioprocessing utilizes saline byproducts such as soybean meal extracts, fish sauce residue, whey permeate, and molasses. Enhancing yeast salt tolerance is therefore critical for reliable and scalable biomanufacturing. Osmolytes are small molecules that stabilize proteins, maintain turgor, and protect cellular integrity, yet their comparative effectiveness in improving *Saccharomyces cerevisiae* performance under food-relevant and industrial stress conditions remains unclear. Here, we present a systematic evaluation of major commercial osmolyte classes under salt stress. *Myo*-inositol, sucrose, and lactose significantly improved yeast growth, viability, and oxidative stress tolerance, supporting more robust and efficient bioprocessing using circular bioresources.

## INTRODUCTION

Yeast fermentation plays a foundational role in numerous industrial sectors, including the production of alcoholic beverages (brewing and wine making), baked goods, bioethanol, functional food, and various high-value biochemicals (1, 2). The fermentative capabilities of *Saccharomyces cerevisiae* enable efficient conversion of sugars into ethanol and carbon dioxide, accompanied by the formation of aroma and flavor compounds that are essential for product quality (3). Beyond food and beverages, *S. cerevisiae* is widely employed as a platform for biopharmaceuticals, enzyme production, metabolic engineering, and other bioactive metabolites of commercial importance (4). Among industrial microorganisms, *S. cerevisiae* stands out for its robustness, generally recognized as safe (GRAS) status, and well-mapped genetics, making it a preferred host for large-scale fermentation and industrial biotechnology worldwide (5–7).

Many industrial processes expose yeast to osmotic stress caused by elevated concentrations of sugars or salts, particularly in high-gravity brewing, sweet dough fermentation, and saline biotechnological processes (8–10). Hyperosmotic conditions cause rapid water efflux from the cytoplasm, leading to decreased turgor pressure, cell shrinkage, metabolic perturbations, and reduced viability (11). In environments containing ~6% or 1.03 M NaCl or equivalent osmolarity, uncontrolled stress responses can severely compromise fermentation yields and process efficiency (12, 13).

A key adaptive strategy of yeast under hyperosmotic stress is the synthesis or accumulation of compatible solutes, small organic molecules known as osmolytes that counterbalance external osmotic pressure without disrupting cellular biochemistry (14). Endogenous osmolyte accumulation (Fig. S1), primarily via the high-osmolarity glycerol (HOG) pathway, helps maintain intracellular hydration and stabilize macromolecular structures (11).

Under high-salt stress, due to the concentration gradient, Na^+^ ions move into the cell, triggering activation of the HOG pathway and stimulating vacuolar ATPase (V-ATPase) activity (15). To counter osmotic stress, yeast cells expend ATP to drive V-ATPase, which facilitates the sequestration of Na^+^ into vacuoles, thereby lowering its cytosolic toxicity (16). Concurrently, the HOG pathway enhances the transcription of genes involved in glycerol (endogenous osmolyte) biosynthesis, which helps in maintenance of cellular integrity and membrane stability (17, 18). This pathway also induces Na^+^ efflux transporters that expel excess Na^+^ ions back into the medium (15). Collectively, these physiological and genetic adaptations reallocate energy resources toward stress tolerance, resulting in lower cellular growth and reproduction (19).

Exogenous supplementation with osmolytes can further enhance stress tolerance, shorten lag phases, and sustain product formation under extreme conditions (20–22). Mechanistically, osmolytes act as molecular chaperones, stabilizing protein folding, lipid bilayer integrity, and enzyme activities during stress (23). Industrial applications include improved dough leavening in baking, enhanced resilience in high-sugar or saline fermentations, and greater robustness of biocontrol yeasts (8, 24). While glycerol is the primary endogenous osmolyte in *S. cerevisiae*, secondary or supplemental osmolytes also contribute to stress protection: trehalose stabilizes membranes and proteins; sorbitol and mannitol act as polyols with osmotic and redox functions (25); proline serves as both an amino acid chaperone and ROS scavenger; xylitol provides antioxidative benefits; and *myo-*inositol functions as a precursor for membrane biosynthesis. Other compounds such as sucrose, lactose, glycine, β-alanine, and urea have demonstrated more limited or condition-specific benefits, often constrained by metabolic compatibility or toxicity thresholds.

From an industrial perspective, adoption of osmolytes is strongly influenced by cost and availability. Commodity solutes such as glycerol and sorbitol (~$1 to $7.44/kg) are widely used in large-scale processes, whereas specialty osmolytes like trehalose, *myo-*inositol, and xylitol can exceed ~$15 to $35/kg (26). High-cost amino acid osmolytes, such as proline, are typically reserved for high-value applications where their performance benefits justify the expense. Thus, pricing directly influences adoption strategies, particularly in large-scale fermentations where material costs must be balanced against productivity gains. Despite their benefits, commercial osmolytes are not without limitations. Over-supplementation can cause metabolic imbalances, undesirable flavor attributes, or excessive osmotic pressure beyond tolerance limits (20, 23). Moreover, most previous studies have evaluated osmolytes individually, often using different strains or experimental conditions, making direct comparisons difficult. To date, no published study has systematically assessed the relative efficacy of a diverse range of commercial osmolytes under standardized high-salt (6% NaCl, ≈1.03 M) fermentation conditions in *S. cerevisiae*. This work addresses that gap by directly comparing representative polyols, sugars, amino acids, and nitrogenous compounds in terms of their ability to support yeast growth, biomass productivity, budding frequency, and morphological recovery under severe salt stress, with implications for industrial applications.

While several studies have explored the mechanistic basis of osmotic adaptation in *S. cerevisiae* through analyses of ATPase activity and ion flux regulation, the present study was designed with a complementary objective. Rather than probing molecular mechanisms, our focus was to establish a comparative, application-oriented framework for evaluating the osmoprotective potential of 12 commercially relevant (low-cost food-grade) osmolytes under standardized high-salt stress conditions. This practical, physiology-based approach provides quantitative insight into growth kinetics, viability, redox balance, and morphological recovery, metrics that are directly translatable to industrial high-osmolarity fermentation processes.

## MATERIALS AND METHODS

### Materials

All chemicals and reagents used in this study were of analytical grade. Commercial osmolytes tested included glycine (≥99% purity, Sigma-Aldrich), L-proline (≥99%, Aldrich), β-alanine (A grade, Calbiochem), D-sorbitol (≥98%, Sigma), D-mannitol (Difco Laboratories), *myo-*inositol (≥99%, Sigma), glycerol (≥99%, Sigma-Aldrich), urea (Sigma), sucrose (ACS certified, Fisher Chemical), anhydrous lactose (Spectrum Chemicals), trehalose dihydrate (Sigma), and xylitol (≥99%, Sigma-Aldrich). Additional reagents used for biochemical assays included ninhydrin (2,2-dihydroxy-1,3-indanedione; Sigma) for amino acid estimation and standard analytical grade reagents for the 2,2-diphenyl-1-picrylhydrazyl (DPPH) radical scavenging assay and Anthrone carbohydrate assay. Sodium chloride (NaCl, ACS grade; Fisher Scientific) was used to induce osmotic stress in yeast cultures. All chemicals were handled according to manufacturer recommendations, and freshly prepared solutions were used for each experiment.

### Experimental method

#### Yeast strain and culture conditions

*Saccharomyces cerevisiae* CBS 1171 was selected for all experiments because of its widespread use in fermentation research and industrial biotechnology (8, 27). Cultures were grown in yeast extract, peptone, dextrose (YPD) medium supplemented with 6% (wt/vol) NaCl to induce osmotic stress, a concentration known to disrupt ion homeostasis and cellular water balance in yeast (28).

#### Experimental design for osmolyte supplementation

Commercial osmolytes from multiple classes of compatible solutes were evaluated for their ability to mitigate salt-induced stress (Table 1). Most osmolytes were supplemented into the culture medium at 1% and 2% (wt/vol), whereas urea was tested at lower levels (0.03% and 0.07% (wt/vol) based on compatibility range in yeast (29).

**TABLE 1:** Commercial osmolytes employed for osmoprotection

Category	Osmolytes
Sugars	Sucrose, trehalose, lactose
Sugar alcohols	Sorbitol, D-mannitol, xylitol, *myo-*inositol
Amino acids	L-Proline, glycine, β-alanine
Polyols and others	Glycerol, urea

#### Growth kinetics and optical density measurements (OD_600_)

Growth kinetics of *S. cerevisiae* under osmotic stress were monitored spectrophotometrically by measuring optical density at 600 nm (OD_600_). *S. cerevisiae* cultures were grown under microaerobic conditions in sterile 52.5 mL polypropylene tubes containing 20 mL of YPD medium as the working volume. This configuration provided a headspace-to-liquid ratio of 1.625:1. Cultures were inoculated to an initial OD_600_ of 0.1–0.2 and incubated at 30°C with continuous agitation at 150 rpm in an orbital shaker to maintain consistent oxygen transfer rates for up to 98 h. Growth was recorded at ~8 h intervals, while final biomass accumulation was determined at 108 h by measuring dry cell weight. Growth curves were used to identify lag, exponential, and stationary phases and to calculate the maximum specific growth rate (µmax) as described in Determination of Specific Growth Rate. Two control conditions were included throughout the study: control (–), yeast grown in standard YPD medium without NaCl (no osmotic stress, no osmolytes); and control (+), yeast grown in YPD medium with 6% NaCl but no osmolytes (osmotic stress control). This design enabled systematic comparison of osmolyte classes in terms of their effects on growth, viability, and stress tolerance under salt stress.

#### Determination of specific growth rate

The specific growth rate (µmax) of *S. cerevisiae* under each osmolyte treatment was determined from growth kinetics measured as OD_600_. The exponential phase of growth was identified by plotting the natural logarithm of OD_600_ against time, and µmax (h⁻¹) was calculated from the slope of the linear regression fit to the exponential portion of the growth curve, and doubling time (Td) was essentially derived from the specific growth rate, as described previously (30), using the formula


$$\begin{array}{c} Specific\;growth\;rate\;(\mu )=(\ln({OD}_{2})-\ln({OD}_{1}))\;/\;(t_{2}-t_{1})\\ \%\;Improvement=(Experimental\;rate-Control\;rate)\;/\;Control\;rate\times 100\\ Td=\ln(2)\;/\;\mu max \end{array}$$

where OD_1_ and OD_2_ are the optical density values at times *t*_1_ and *t*_2_ within the exponential growth phase. Data were analyzed in triplicate, and the mean ± standard deviation (SD) was reported.

#### Viability assessment by colony-forming units

Cell viability under osmotic stress was assessed by colony-forming unit (CFU) enumeration. At the end of the fermentation, culture aliquots were serially diluted to 10^−5^ and 10^−6^ and plated on YPD agar. Plates were incubated at 30°C for 24 h, after which colonies were counted manually and expressed as CFU per milliliter of culture (×10⁶ CFU/mL). Relative viability was calculated as the percentage change in CFU compared to the stressed control (6% NaCl without osmolyte), thereby quantifying the osmoprotective effects.

#### Budding cell and morphological analysis

Morphological characterization of *S. cerevisiae* under osmotic stress was conducted by light microscopy. At 48 h, culture aliquots were harvested, mounted on glass slides, and observed using a bright-field microscope (EVOS; Thermo Fisher Scientific, USA) at 200× magnification. Cells were classified manually as budding cells (cells with visible bud formation) or non-budding (single cells), and counts were expressed as percentages of the total cell population. Total cell density (×10⁶ cells/mL) was determined using a hemocytometer. All analyses were performed in triplicate. In addition, morphological features, such as cell size and budding frequency, were recorded to evaluate the effect of osmolyte supplementation on growth physiology under 6% NaCl stress.

#### Yeast cell diameter measurement

Cell size analysis was performed to assess the effect of osmolyte supplementation on yeast morphology under salt stress. At 48 h, cells were harvested and imaged using a bright-field microscope at 200× magnification. Digital images were captured and analyzed with ImageJ software (NIH, USA) following standard protocols. Scale calibration was performed using a stage micrometer, and the diameter of at least 200 cells per treatment was measured to ensure statistical robustness. The mean cell diameter (µm) and SD were calculated for each treatment. Comparisons were made between the unstressed control (0% NaCl), stressed control (6% NaCl), and osmolyte-supplemented groups to assess osmoprotective effects on cell integrity and morphological adaptation under hyperosmotic conditions.

#### Biochemical assays

Biochemical assays were conducted to quantify metabolites and stress-related markers in *S. cerevisiae* under osmotic stress and osmolyte supplementation. Samples were collected at 0 h (pre-fermentation) and 98 h (post-fermentation), centrifuged at 5,000 × *g* for 10 min, and the resulting supernatants were used for analysis. All assays were conducted in triplicate, and results are reported as mean ± SD.

##### Estimation of total reducing sugar consumption

Total reducing sugars were quantified using the 3,5-dinitrosalicylic acid (DNS) method with glucose as the standard (31). Culture aliquots collected at 0 and 98 h were centrifuged to remove cells, and clarified supernatants were diluted to fall within the linear range of the assay (≤1.0 mg/mL glucose equivalents). Equal volumes of sample (or standard) and DNS reagent were mixed in screw-cap tubes, heated at 95°C–100°C for 5–10 min, and rapidly cooled, and the absorbance was read at 540 nm using a microplate spectrophotometer (Epoch 2; Biotech, USA) against reagent blanks. Glucose concentrations were determined by linear regression of the standard curve (*R*² ≥ 0.98), corrected for dilution, and expressed as milligram per milliliter glucose equivalents. Net sugar consumption was calculated as Δ = *C*_0h_ − *C*_98h_. All measurements were performed in technical replicate (≥2), and matrix blanks were included where appropriate.

##### Antioxidation capacity: radical scavenging assay

The antioxidant capacity of *S. cerevisiae* cultures subjected to osmotic stress (6% NaCl) was assessed using the DPPH radical scavenging assay with minor modifications (32, 33). Assays were conducted at two time points: prior to fermentation (0 h) and after 98 h of fermentation. Cell-free extracts were obtained by centrifuging culture samples at 8,000 × *g* for 5 min, and the supernatants were collected. A 1 mL aliquot of each extract was mixed with 2 mL of freshly prepared 0.2 mM DPPH solution in ethanol. The mixtures were incubated in the dark at room temperature for 30 min, and absorbance was recorded at 517 nm using a microplate spectrophotometer (Epoch 2; BioTek, USA). Ethanol served as the blank, while a DPPH solution without yeast extract served as the control.

The radical scavenging activity (%RSA) was calculated using the following equation:


$$\%RSA=(1-A_{control}/A_{sample}\times 100),$$

where *A*_sample_ is the absorbance of the sample with DPPH, and *A*_control_ is the absorbance of ethanol with DPPH without the sample.

##### Estimation of total free amino acids

The total free amino acid content of *S. cerevisiae* under high salt stress was determined using the ninhydrin colorimetric method, with glycine as the reference standard (34). Cultures supplemented with different osmolytes were sampled at two points: prior to fermentation (0 h) and after 98 h of fermentation. For the assay, 100 µL of each supernatant was mixed with 100 µL of freshly prepared 0.2% ninhydrin reagent in citrate buffer (pH 5.5). The reaction mixtures were incubated at 100°C for 15 min to allow for chromophore development, followed by cooling to room temperature. Absorbance was measured at 570 nm using a microplate spectrophotometer (Epoch 2, BioTek). A standard calibration curve was prepared using glycine solutions (0–100 μg/mL), and free amino acid concentrations were expressed as milligram glycine equivalents per milliliter of culture. Each treatment was analyzed in triplicate, and results were reported as mean ± SD. Data were normalized to sample volume and presented as percentage changes in free amino acids between 0 and 98 h. Unstressed and salt-stressed controls without osmolyte supplementation were included for comparison.

##### Determination of total carbohydrate content

The total carbohydrate content of culture supernatants was determined using the Anthrone method (35) with minor modifications. Samples were collected at 0 h (pre-fermentation) and 98 h (post-fermentation) and centrifuged at 5,000 × *g* for 10 min to remove yeast biomass, and the clarified supernatants were used for analysis. Anthrone reagent was freshly prepared by dissolving 0.2 g Anthrone in 100 mL concentrated sulfuric acid, cooled on ice, and protected from light until use. For each assay, 200 µL of culture supernatant was mixed with 1 mL of Anthrone reagent, vortexed, and heated in a boiling water bath at 95°C for 10 min to allow color development. After cooling to room temperature, absorbance was measured at 620 nm using a microplate spectrophotometer (Epoch 2, BioTek). Glucose was used as the standard for calibration, and carbohydrate concentrations were expressed as milligram per milliliter of culture supernatant. All assays were performed in triplicate, and results are reported as mean ± SD. The relative change in carbohydrate concentration (Δ, %) between 0 and 98 h was calculated to evaluate sugar utilization efficiency under osmolyte supplementation during salt stress.

### Yeast biomass determination

Yeast biomass production was quantified as dry cell weight (g/L, dry basis). After 98 h of fermentation, culture broth was collected and cells were harvested by centrifugation at 4,500 × *g* for 10 min at 4°C. The resulting yeast pellet was washed with distilled water to remove residual medium components. The washed pellet was then dried to constant weight in a hot air oven at 80°C for 48 h. Biomass concentration was expressed as gram per liter (DB) by normalizing the dry weight to the culture volume.

### Multivariate assessment: principal component analysis and heatmap clustering

To evaluate global trends and treatment-specific clustering of biochemical and growth responses, principal component analysis (PCA) and hierarchical clustering with heatmap visualization were performed. All quantitative data sets, including OD_600_ values, colony-forming units, dry cell weights, budding cell percentages, cell diameter, total carbohydrates, reducing sugars, amino acids, and antioxidant capacity, were compiled into a single data matrix. Each variable was normalized using *z*-score transformation to ensure comparability across assays.

PCA was conducted on the covariance matrix to extract principal components (PCs) that explained the maximum variance within the data set. The first two principal components (PC1 and PC2) were used for visualization to highlight separation among osmolyte treatments and stressed controls (6% NaCl). Heatmap analyses were performed to visualize treatment-specific groupings and correlation patterns among osmolytes. All PCA and clustering analyses were carried out using Minitab (36).

### Data analysis

Results are reported as mean ± SD. The percentage change in reducing sugars between 0 and 98 h was calculated for each treatment. Statistical significance was assessed using one-way ANOVA followed by Tukey’s HSD post hoc test, with a threshold of *P* < 0.05.

## RESULTS

### Determination of optimal salt stress concentration for osmotic stress induction

To determine the optimal NaCl concentration for inducing osmotic stress in *S. cerevisiae* without completely inhibiting growth, yeast growth was monitored for optical density (OD_600_) over time across a range of NaCl levels (0%–8%). Growth curves (Fig. S2) demonstrated concentration-dependent inhibition, with unstressed cultures (0% NaCl) exhibiting rapid exponential growth, reaching a maximum OD of 7.224 at 66 h and a specific growth rate of 0.18 h⁻¹ during the early exponential phase (4–22 h). Low NaCl concentrations (0.5%–2%) showed minimal impact, with maximum ODs ranging from 6.248 to 7.144, indicating tolerance to mild osmotic pressure, consistent with yeast’s adaptive mechanisms like the HOG pathway (8, 37). At 4% NaCl, growth was moderately impaired, with a maximum OD of 5.684 (21.3% reduction relative to 0% NaCl) and a growth rate of 0.14 h⁻¹ (19.1% reduction). A pronounced inhibitory effect was observed at 6% NaCl, where the maximum OD decreased to 4.732 (34.5% reduction) and the growth rate dropped sharply to 0.029 h⁻¹ (83.7% reduction), reflecting significant osmotic stress but still permitting measurable proliferation. In contrast, 8% NaCl nearly abolished growth, with a maximum OD of 0.428 (94.1% reduction) and a negative growth rate (0.003 h⁻¹), suggesting cellular arrest or viability loss (38), and similar trends were also reported by Nasser (39).

Based on these findings, 6% NaCl was selected as the stress condition for subsequent experiments, as it imparts substantial osmotic stress evidenced by extended lag phases, reduced growth rates, and lower stationary-phase densities while allowing sufficient cell survival and metabolic activity to evaluate osmoprotectant effects. This concentration balances stress induction with experimental feasibility, enabling systematic assessment of osmolyte-mediated restoration of growth and physiology.

### Impact of osmotic stress on cell growth

Microbial cultivation under industrial conditions often deviates from the controlled environment of synthetic laboratory media. To establish a baseline for stress response, the growth of *S. cerevisiae* was first examined under unstressed and salt-stressed conditions in the absence of osmolytes. The unstressed control (0% NaCl, no osmolyte) exhibited typical logistic growth, reaching a final OD_600_ of 1.193 ± 0.013 after 98 h, consistent with stationary-phase entry in rich medium. In contrast, exposure to 6% NaCl resulted in a markedly extended lag phase, with OD_600_ values remaining near 0.08–0.09 through 24–38 h and visible growth only resuming after ~62 to 75 h. Final cell density reached just 0.346 ± 0.016 OD_600_, a 71% reduction relative to the control, confirming that high salinity imposes severe osmotic stress and significantly compromises proliferation. This stress model is highly relevant when considering the application of industrial byproducts (e.g., soy molasses, distillers’ solubles, and fruit-processing residues) for microbial cultivation, which often contain elevated salt levels that restrict microbial growth and limit their direct use as fermentation substrates (40–42).

Establishing the inhibitory impact of 6% NaCl therefore provides a realistic benchmark for assessing whether osmolyte supplementation can mitigate stress and restore growth performance. To assess whether osmolytes could mitigate these effects, the salt-stressed medium (YPD + 6% NaCl) was supplemented with commercial osmolytes at 1% or 2% (wt/vol), or at 0.03% and 0.07% for urea due to potential toxicity. Growth parameters were extracted from OD_600_ data, with μmax calculated using an interval-based method and lag phase defined as the time to reach 1.5× the initial OD_600_.

In the following sections, the osmoprotective effects of 12 different commercial osmolytes are evaluated, with the goal of identifying solutes that enhance yeast tolerance under such challenging conditions.

### Effect of osmolytes on yeast growth at high osmotic stress

**Fig 1:**
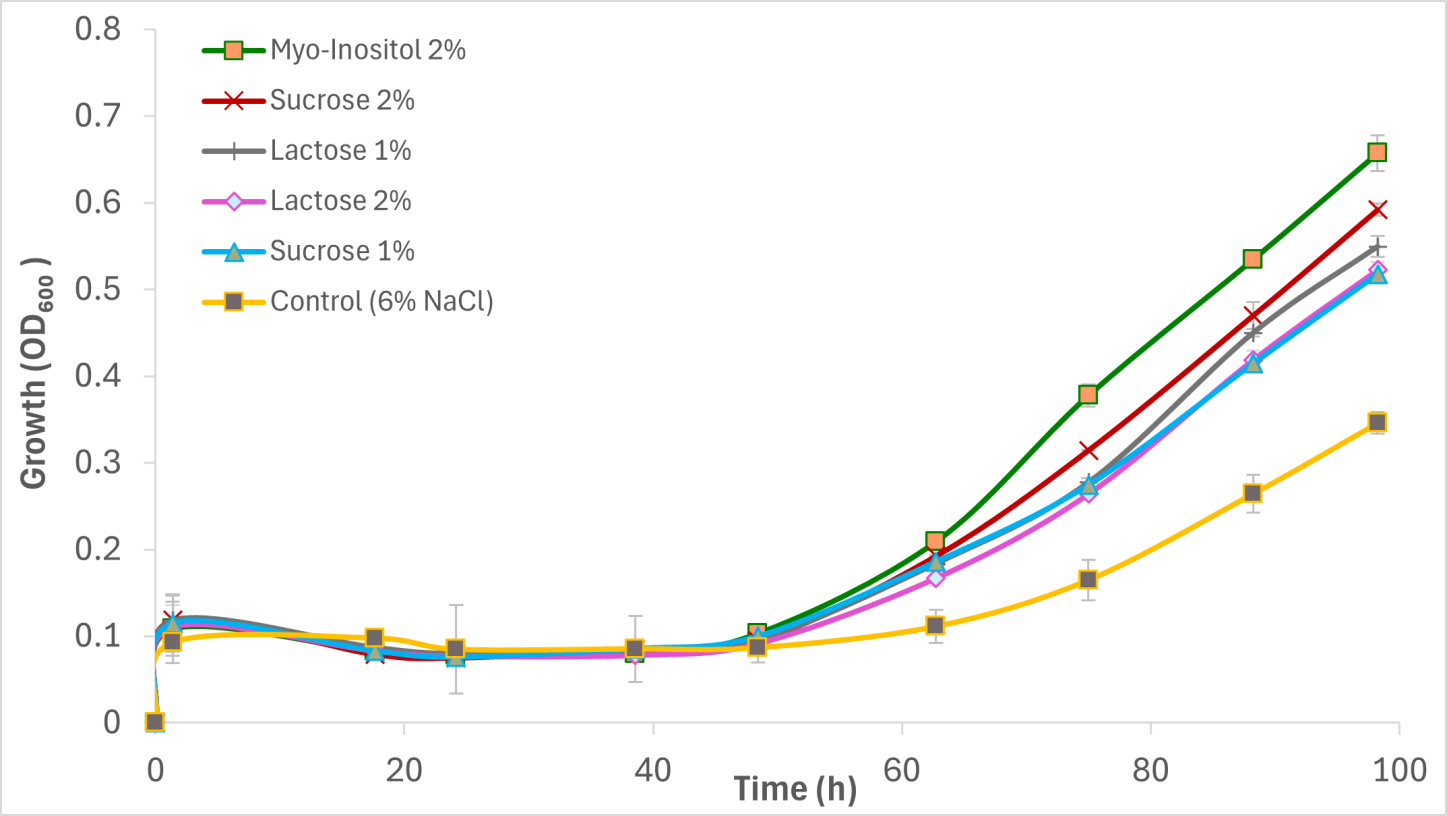
Comparison of growth kinetics for the top five osmoprotectants in *S. cerevisiae* under high salt stress.

Cultures supplemented with certain osmolytes resumed growth earlier and achieved significantly higher densities than the unsupplemented 6% NaCl control (+) (Fig. 1; Fig. S3 and S4). By 98 h, supplementation alleviated the inhibitory effects of NaCl in most cases, leading to improved growth kinetics and higher final cell densities (means of triplicates ± SD). Based on overall effectiveness across growth, viability, and biomass metrics, osmolytes were grouped into four categories: strong osmoprotection (*myo-*inositol, sucrose, and lactose), moderate osmoprotection (trehalose, sorbitol, glycerol, xylitol, and mannitol), and minimal osmoprotection (glycine, proline, β-alanine, and urea). To quantify and compare the osmoprotective effects, specific growth rates (*μ*) and percentage improvement over the stressed control were calculated during the active growth phase (48–88 h) (Table S1).

#### Osmolyte class I: disaccharides (sucrose, trehalose, and lactose)

**Fig 2:**
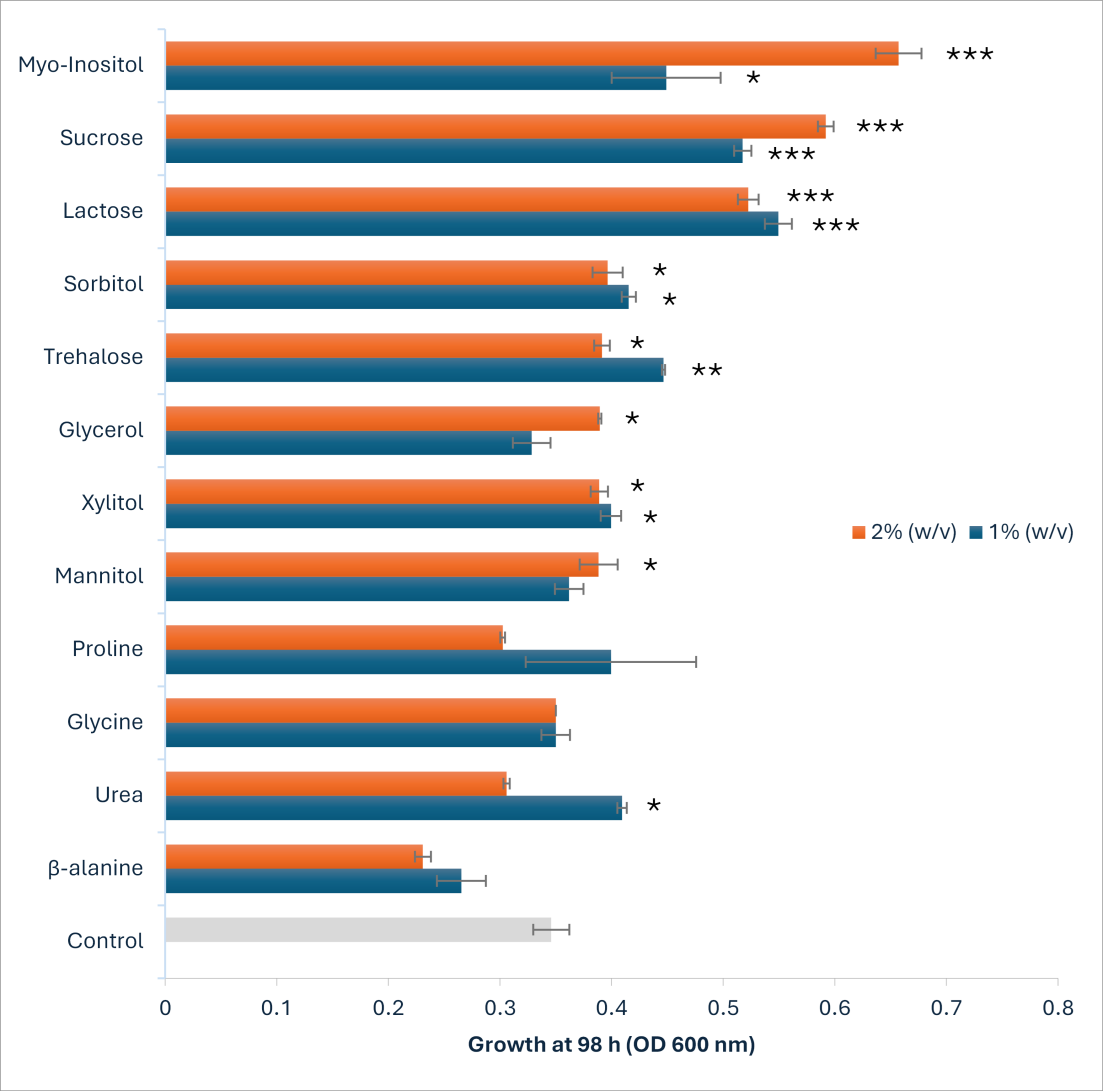
Comparative final growth (OD_600_) of *S. cerevisiae* under 6% NaCl with 12 different osmolytes at 1% and 2% (wt/vol). Data represent mean ± SD (*n* = 3). Statistical significance relative to the salt-stressed control (+) is indicated by asterisks: *, *P* < 0.05; **, *P* < 0.01; ***, *P* < 0.001.

Among the disaccharides tested, sucrose exhibited strongest osmoprotective effects. At 2% supplementation, sucrose increased μmax by 79.8% (to 0.0497 h⁻¹, *P* < 0.01) and final OD_600_ by 71.1% (to 0.592, *P* < 0.001) while reducing in lag phase by 12.4% (to 60 h, *P* < 0.05) (Fig. 2; Fig. S5). At 1%, sucrose still produced substantial improvements, with final OD_600_ enhanced by 49.6% (to 0.5175, *P* < 0.001) and μmax by 21.7% (to 0.0434 h⁻¹, *P* < 0.05), alongside a similar reduction in lag duration (12.5%). These findings suggest that sucrose may function both as an extracellular osmolyte and as a metabolizable carbon source. However, further studies are required to determine whether it contributes to intracellular glycerol accumulation through stress-responsive metabolic pathways. Lactose showed comparable protective effects. At 1%, the final OD_600_ increased by 58.8% (to 0.5495, *P* < 0.001) and μmax by 27.6% (to 0.0455 h⁻¹, *P* < 0.01), while lag was reduced by 10.9% (to 61.62 h, *P* < 0.05). Interestingly, the 2% treatment was slightly less effective (final OD_600_ +51.0%, μmax +18.1%), likely reflecting the limited capacity of *S. cerevisiae* to hydrolyze lactose due to its lack of native β-galactosidase activity. Trehalose, a well-known stress protectant and membrane stabilizer, produced moderate improvements at 1% (final OD_600_ +29.0% to 0.4465, μmax +26.7% to 0.0451 h⁻¹; both *P* < 0.05). However, its effects diminished at 2% (final OD_600_ +13.2%, μmax +17.1%), with only marginal reductions in lag phase (1.6%–2.1%). This pattern is consistent with the literature, suggesting that trehalose may function primarily through protein stabilization and ROS scavenging, rather than through direct osmotic balancing in this specific stress model.

#### Class II: sugar alcohols and polyols (sorbitol, mannitol, *myo-*inositol, xylitol, and glycerol)

Polyols exhibited the broadest range of efficacy, with *myo-*inositol standing out as the most potent. At 2%, *myo-*inositol increased final OD_600_ by 90.0% (to 0.657, *P* < 0.001) and μmax by 38.5% (to 0.0494 h⁻¹, *P* < 0.01) and shortened lag duration by 18.4% (to 56.43 h, *P* < 0.01). The 1% concentration was nearly as effective, improving final OD_600_ by 29.8% (μmax +4.4%) and reducing lag by 21.2%, highlighting its potential role as a precursor for sphingolipid synthesis and its likely contribution to maintaining membrane integrity under osmotic stress. Sorbitol provided moderate protection at 1%, increasing final OD_600_ by 20.1% (to 0.4155, *P* < 0.05), though improvements in μmax (+4.4%) and lag time (−0.7% extension) were minimal. At 2%, sorbitol was less effective overall. Mannitol provided minimal benefits, with only modest increases in final OD_600_ (+4.6 to12.3%) and slight reductions in μmax (−12.0% to −7.3%). The limited response suggests lower osmoprotective efficacy under these conditions; however, the underlying mechanism was not directly investigated in this study. Xylitol showed moderate activity at 1%, raising final OD_600_ by 15.5% and reducing lag by 12.3%, though μmax decreased (−10.6%). At 2%, these protective effects diminished. Glycerol, the native endogenous osmolyte of *S. cerevisiae*, enhanced growth modestly at 2%, with the final OD_600_ increased by 12.6% (*P* < 0.05), lag reduced by 8.6%, but μmax decreased by 9.9%. Overall, *myo-*inositol consistently outperformed the other polyols, reinforcing its dual role as both a compatible solute and a metabolic precursor critical for membrane adaptation under osmotic stress.

#### Class III: amino acids (proline, glycine, and β-alanine) and urea

Amino acids and urea generally provided limited or negative osmoprotection under high salt stress. Proline offered modest benefits at 1%, increasing final OD_600_ by 15.5% (to 0.3995) and shortening lag duration by 13.9% (to 59.53 h). These effects are consistent with the role of proline as a reactive oxygen species scavenger. At 2%, however, proline reduced the final OD_600_ (−12.6%) and μmax (−20.8%), suggesting that excess accumulation may be metabolically burdensome. Glycine showed negligible effects, with minor or inconsistent changes in growth parameters (final OD_600_ +1.2%, μmax +14.0%), often accompanied by extended lag phases. β-Alanine was the least effective, decreasing the final OD_600_ by 23.3%–33.2% and prolonging lag by 10.5% at 2%, likely due to interference with central metabolism. Urea supplementation showed a biphasic effect at 0.03%; final OD_600_ increased by 18.4%, but at 0.07%, OD_600_ decreased (−11.6%), consistent with its dual role as a nitrogen source and potential protein denaturant.

Overall, *myo-*inositol, sucrose, and lactose were the most effective osmolytes, consistently improving yeast performance under 6% NaCl stress. These solutes increased final biomass by more than 50% and growth rates by at least 25% compared to the salt-stressed control. In contrast, β-alanine exacerbated stress, while sorbitol, mannitol, and glycine produced only minimal improvements. Concentration effects were strongly osmolyte specific; polyols and disaccharides often provided greater protection at 2%, whereas amino acids showed diminishing returns.

### Viability assessment via colony-forming units under high salt stress

The viability of *S. cerevisiae* cells was evaluated by CFUs (CFU/mL × 10⁶) at 108 h post-inoculation in YPD medium under 6% NaCl, with or without osmolyte supplementation. Cell viability was strongly impacted by salt stress and varied considerably with osmolyte supplementation (Table S2). Treatments were ranked according to CFU per milliliter, excluding controls to provide a clear baseline. Comparative indices were calculated, with % viability vs control (−) representing the proportion of unstressed viability restored.

The unstressed control (−, C0) maintained the highest viability (81.21 × 10⁶ CFU/mL). In contrast, the salt-stressed control (+, C6) exhibited an ~82% reduction in viability, reaching only 14.54 × 10⁶ CFU/mL (17.9% of C0). Among the osmolytes, disaccharides and polyols showed the greatest protective effects. Lactose 2% restored viability to 21.41 × 10⁶ CFU/mL (26.4%), followed closely by *myo-*inositol 2% (23.4%) and sucrose 2% (21.9%). Xylitol 2% and trehalose 1% also showed moderate benefits (~18% to 19%). By contrast, amino acids, urea, and glycerol provided minimal or negative effects. Proline 2%, glycine, β-alanine, and urea (0.07%) clustered near or below the control (+), while β-alanine 2% showed the lowest viability (4.44 × 10⁶ CFU/mL, 5.5%). Overall, the most effective osmolytes (lactose, *myo-*inositol, and sucrose) enhanced cell viability by 20%–50% relative to the salt-stressed control, while ineffective osmolytes offered little protection. These trends align with biomass and growth data, supporting the conclusion that disaccharides and *myo-*inositol provide superior osmoprotection under high-salt stress.

### Cell imaging and morphological analysis under osmotic stress

Bright-field microscopy at 200× magnification revealed distinct morphological responses of *S. cerevisiae* to osmotic stress and osmolyte supplementation (Fig. S13). The unstressed control (−) displayed typical morphology, with round to oval cells, smooth intact boundaries, and active budding (Fig. S13A). Numerous daughter cells were observed attached to mother cells, reflecting robust cell division and uniform population growth.

In contrast, the salt-stressed control (+) showed pronounced shrinkage, plasmolysis-like collapse, and irregular cell boundaries, reflecting osmotic dehydration and membrane tension (Fig. S13B). Cell diameters were visibly smaller than in the unstressed control, and budding activity was markedly reduced, with many cells appearing isolated and inactive. Supplementation with effective osmolytes partially restored morphology toward the unstressed phenotype. *Myo-*inositol (2%) produced enlarged, spherical cells with restored dimensions (Fig. S13C). Sucrose (2%) improved smoothness and enhanced axial budding, with clusters of actively dividing cells (Fig. S13D). Lactose (1%) reduced the proportion of collapsed cells and promoted bipolar budding, indicative of restored metabolic activity (Fig. S13E). Together, these findings show that osmolyte supplementation alleviates NaCl-induced morphological damage, restores turgor balance, and promotes structural and functional recovery in *S. cerevisiae*.

#### Yeast cell diameter changes under high salt stress

**Fig 3:**
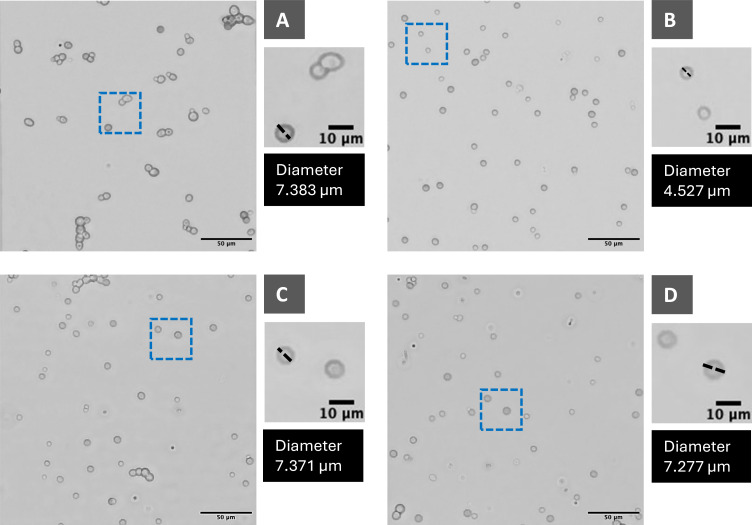
Microscopic analysis of *S. cerevisiae* cell morphology and diameter under salt stress and osmolyte supplementation. (**A**) Unstressed control (7.383 µm), (**B**) NaCl-stressed control (4.527 µm), (**C**) *myo*-inositol 2% supplementation (7.371 µm), (**D**) sucrose 2% supplementation (7.277 µm). Salt stress significantly reduced cell size, whereas osmolyte supplementation restored cell diameter close to unstressed levels.

The unstressed control (−) exhibited an average cell diameter of 7.61 ± 1.03 µm, reflecting normal morphology under optimal growth conditions. In contrast, cells exposed to 6% NaCl showed a significantly reduced diameter of 4.31 ± 0.23 µm (*P* < 0.001), consistent with osmotic shrinkage and loss of turgor pressure (Fig. 3).

**Fig 4:**
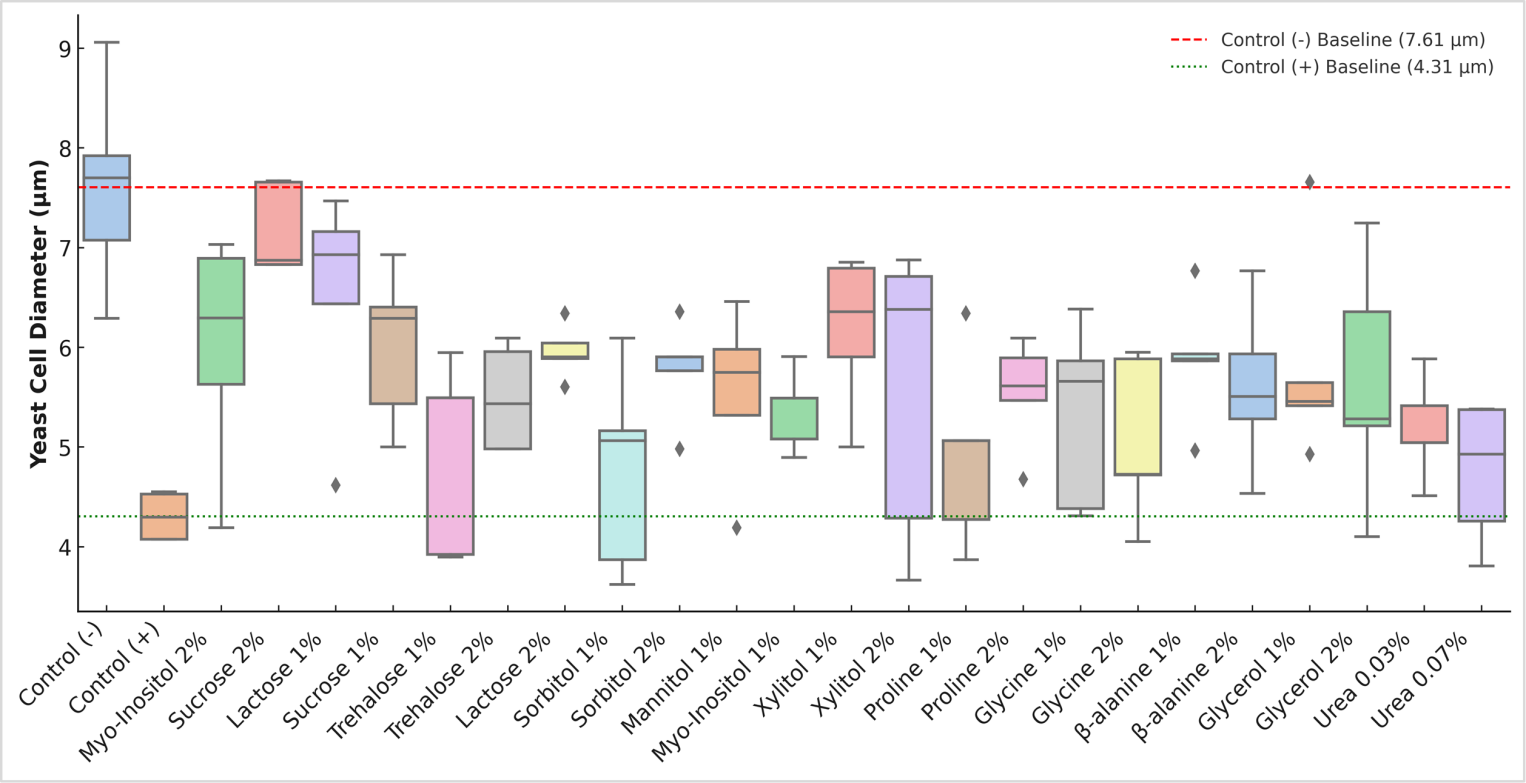
Boxplot of yeast cell diameter distribution under salt stress with various osmolytes.

Supplementation with osmolytes partially restored cell size in a concentration- and type-dependent manner. The most effective treatments were sucrose 2% (7.17 ± 0.45 µm, 66.59% increase; *P* < 0.001), *myo-*inositol 2% (6.80 ± 0.32 µm, 57.88% increase; *P* < 0.001), lactose 1% (6.52 ± 1.13 µm, 51.52% increase; *P* < 0.001), and xylitol 1% (6.18 ± 0.76 µm, 43.60% increase; *P* < 0.001) (Fig. 4; Fig. S6). These values approached or exceeded 6 µm, indicating substantial recovery relative to the stressed control. Notably, sucrose 2% and *myo-*inositol 2% nearly restored diameters to unstressed levels, demonstrating their strong osmoprotective effects under high salt stress. One-way ANOVA revealed highly significant differences in mean cell diameter among treatments (*P* < 0.001), and Tukey’s HSD post hoc test showed that several osmolyte-supplemented groups were significantly different from the salt-stressed control (+).

In contrast, weaker osmolytes, such as urea (0.03%–0.07%) and sorbitol (1%), resulted in only modest improvements, with diameters ranging from 4.7 to 5.6 µm. These treatments failed to fully restore normal morphology, indicating limited osmoprotective capacity under high salt stress.

#### Yeast budding cells under salt stress

**Fig 5:**
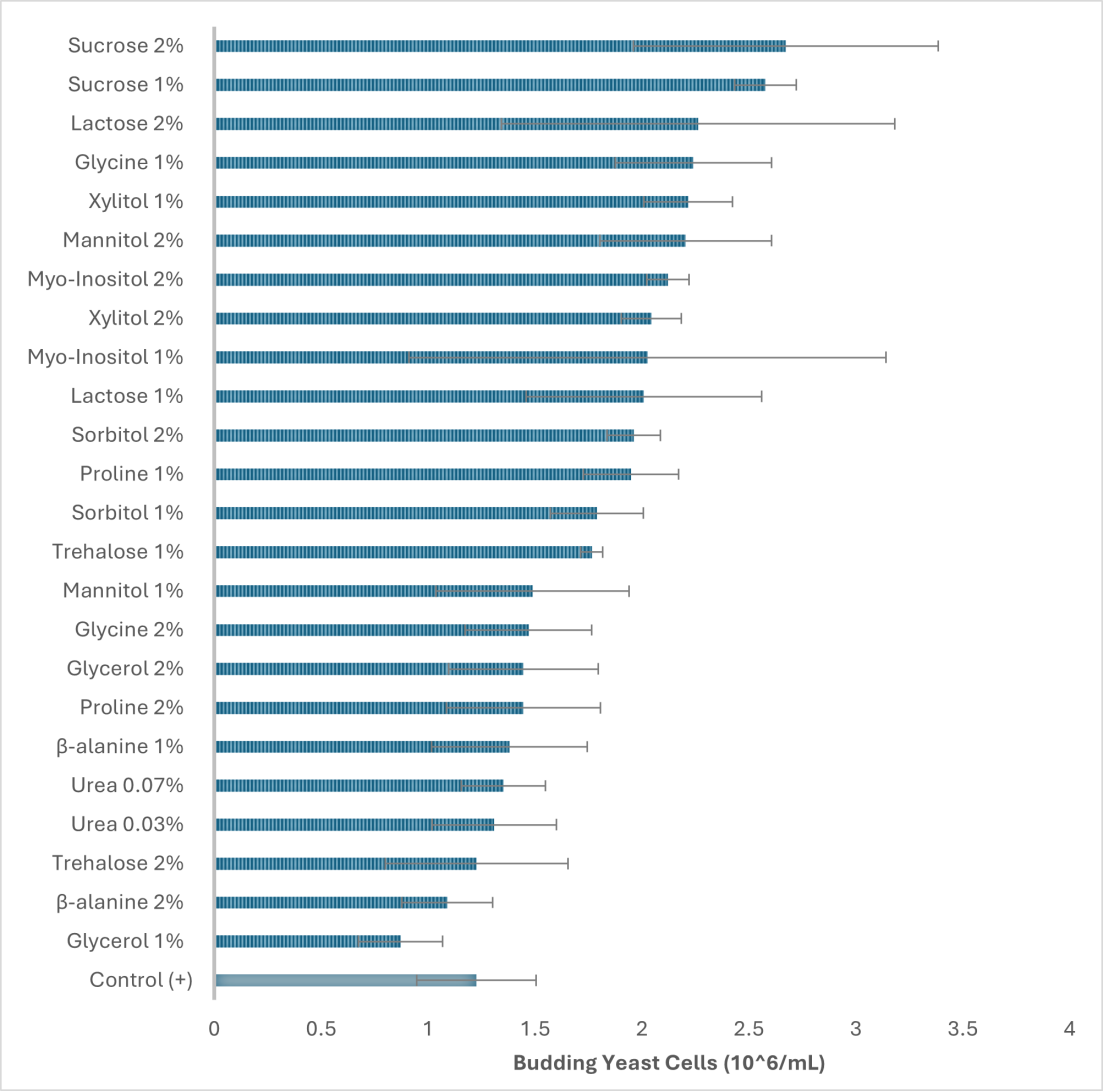
Effect of osmolytes on budding yeast cells under high salt stress (at 48 h).

Cell morphology and population dynamics showed strong effects of salt stress and osmolyte supplementation. The absolute budding cell percentages in *S. cerevisiae* were quantified after 48 h to assess cell division under 6% NaCl. The unstressed control displayed the highest absolute budding frequency (74.76% ± 21.63%), reflecting normal proliferation, whereas the stressed control dropped sharply to 14.42% ± 3.70%, confirming the inhibitory effect of high salt on cell cycle progression (Fig. 5). Osmolyte supplementation partially restored budding activity, though efficacy varied by compound and concentration. Disaccharides (2% and 1%) were most effective; sucrose increased budding by 83.3% (*P* < 0.005) and 77.8% (*P* = 0.005), while lactose improved by 71.3% (*P* = 0.007) and 60.2% (*P* = 0.01), respectively, relative to the salt-stressed control. Similarly, *myo-*inositol also strongly enhanced budding, with increases of 79.9% and 50.6%. Among polyols, xylitol showed the highest stimulation (66.9%–41.2%) (*P* < 0.05), followed by sorbitol (57.7%–54.3%) (*P* < 0.01) and mannitol (62.6%–31.1%) (*P* < 0.05). Amino acid-derived osmolytes exhibited mixed responses. Proline (1%) and glycine (1%) improved budding by ~53%, but higher concentrations were less effective (23%–27%). β-Alanine provided minimal protection, while glycerol and urea were weak or inconsistent, with glycerol showing even negative effects.

#### Non-budding (single) cell counts

Non-budding cells were generally more abundant than budding cells across treatments, indicating a predominance of quiescent or non-dividing populations under stress. The unstressed control exhibited the highest non-budding cell count (141.51 ± 13.46 × 10⁴ cells/mL), whereas the stressed control declined drastically to 6.82 ± 0.30 × 10⁴ cells/mL. Osmolyte supplementation partially alleviated this reduction, with sucrose 1% yielding the highest non-budding count among treatments (8.22 ± 0.56 × 10⁴ cells/mL), followed by trehalose 2% (8.01 ± 0.68 × 10⁴ cells/mL) and *myo-*inositol 2% (7.86 ± 0.35 × 10⁴ cells/mL). In contrast, glycerol 1% (5.94 ± 0.19 × 10⁴ cells/mL) and β-alanine 1% (6.22 ± 0.50 × 10⁴ cells/mL) were the least effective, suggesting limited protection of non-dividing populations.

#### Total yeast cell counts

Total yeast cell counts (10⁶ cells/mL) varied widely across treatments, from 6.95 ± 0.70 glycerol 1% to 141.51 ± 13.46 in the unstressed control (−). The stressed control (+) exhibited a markedly reduced count of 8.31 ± 0.74 × 10⁶ cells/mL, highlighting the inhibitory effect of salt stress. Osmolyte supplementation generally enhanced total counts relative to the stressed control (+). Among treatments, sucrose 1% (10.57 ± 0.95 × 10⁶ cells/mL, +27.2%), trehalose 2% (10.71 ± 0.96 × 10⁶ cells/mL, +28.9%), and *myo-*inositol 1% (10.34 ± 0.93 × 10⁶ cells/mL, +24.4%) were most effective. Conversely, glycerol 1% (6.95 ± 0.70 × 10⁶ cells/mL, –16.4%) and β-alanine 1% (8.06 ± 0.72 × 10⁶ cells/mL, –3.0%) were the least effective, suggesting limited or negative contributions to cell proliferation under salt stress.

### Biochemical evaluation

#### Sugar consumption analysis under salt stress conditions

The analysis of sugar consumption patterns revealed substantial differences in metabolic recovery among the tested osmolyte treatments compared to salt-stressed controls. Reducing sugar content was measured using the DNS method at 0 h (pre-fermentation) and 98 h (post-fermentation) to assess metabolic activity under 6% NaCl stress. At 0 h, baseline values were generally consistent across treatments at approximately 17–18 mg/mL, comparable to the stressed control (17.67 ± 0.07 mg/mL), except for lactose treatments where initial levels were elevated due to its reducing nature (29.31 ± 0.61 mg/mL for 1% and 33.56 ± 0.60 mg/mL for 2%) (Fig. S7). Under 6% NaCl stress conditions, the control (+) demonstrated limited sugar utilization, consuming only 33.7% of available glucose over the 98-h fermentation period, compared to 97% consumption by the unstressed control (−).

Osmolyte supplementation altered this trend substantially. Sucrose (2%) treatment uniquely resulted in a net increase in reducing sugars (+18.4%), likely due to excess invertase-mediated hydrolysis exceeding uptake capacity, whereas sucrose 1% was efficiently consumed. Trehalose (1% and 2%) displayed strong sugar depletion (–43.4% and –37.2%, respectively), reflecting enhanced metabolic consumption. *Myo-*inositol emerged as the most effective, showing strong concentration-dependent effects. *Myo-*inositol 2% treatment enabled sugar consumption of 83.7%, representing a 2.5-fold improvement over the control (+). Even *myo-*inositol 1% supported 55.7% consumption, demonstrating 1.6-fold enhancement over the control. Conversely, β-alanine (2%), urea (0.07%), and glycerol (1%–2%) maintained sugar levels similar to the stressed control, reflecting minimal metabolic improvement. Collectively, treatments with *myo-*inositol (2%), trehalose (1%–2%), and lactose (2%) promoted the most efficient sugar utilization, while sucrose (2%) behaved differently, leading to net sugar accumulation.

#### Carbohydrate dynamics under salt stress

The carbohydrate content of the culture medium, expressed as milligram per milliliter glucose equivalents, was quantified at 0 h (pre-fermentation) and 98 h (post-fermentation) using the Anthrone method. Initial carbohydrate concentrations ranged from 17.94 mg/mL in the stressed control to 46.82 mg/mL in lactose 2%, reflecting differences in supplementation (Fig. S8). After 98 h, all treatments showed reductions in carbohydrate levels, consistent with sugar utilization by yeast.

In the controls, the unstressed culture (−) exhibited near-complete depletion, declining from 21.85 to 1.02 mg/mL (–95.3%), whereas the stressed control (+) showed only partial utilization, decreasing from 17.94 to 8.97 mg/mL (–50.0%), indicative of impaired sugar consumption under osmotic stress. Osmolyte supplementation altered these trends. Sucrose 2% (–31.5%) and trehalose 1% (–30.8%) retained the highest carbohydrate levels, suggesting slower utilization compared with the stressed control. In contrast, *myo-*inositol 2% (–93.6%) and mannitol 2% (–80.1%) promoted extensive consumption, with depletion approaching that of the unstressed control. Lactose treatments showed intermediate effects, with lactose 2% retaining ~42.9% of its initial carbohydrates and lactose 1% retaining ~38.5%. Overall, carbohydrate utilization under salt stress was strongly osmolyte dependent. Disaccharides such as sucrose and trehalose slowed consumption, acting as protective reserves, while polyols such as *myo-*inositol and mannitol were rapidly metabolized, driving higher carbohydrate turnover despite the saline environment.

#### Antioxidant capacity under salt-induced oxidative stress

The DPPH assay further revealed differences in redox balance. The unstressed control increased its %RSA from 53.1% to 61.6% over 98 h, indicating induction of antioxidant defenses during fermentation (Fig. S9). In contrast, the stressed control declined to 39.2% (−18.6%), reflecting ROS accumulation under high salt. Osmolyte supplementation produced divergent effects. Glycine (2%) yielded the strongest antioxidant response (57.6%, +47% vs control [+]), followed by β-alanine, sucrose, lactose, and proline, all of which maintained or enhanced RSA compared with stressed control. Conversely, sorbitol, mannitol, trehalose, glycerol, and urea showed weak or negative effects, with RSA values lower than the stressed control. Concentration-dependent effects were evident, with 2% generally outperforming 1% for glycine (+26%), β-alanine (+4%), and sucrose (+3.7%). However, higher concentrations reduced efficacy for other osmolytes, including *myo-*inositol (−15.9%) and polyols. Together, these findings indicate that osmolytes not only modulate growth and morphology but also impact metabolic and antioxidant responses. Effective osmolytes such as *myo-*inositol, lactose, and sucrose improve carbon utilization and support redox balance, whereas others (β-alanine and urea) may exacerbate oxidative stress or provide minimal benefit.

#### Extracellular amino acid dynamics under salt stress

Free amino acids (primary amines) levels in the medium before and after 98 h of fermentation further reflected metabolic adjustments to salt stress. At 0 h, concentrations ranged between 1.68 and 2.75 mg/mL across treatments (Fig. S10).

In the controls, amino acids (glycine equivalent concentration) remained relatively stable, decreasing slightly in the unstressed culture (−3.4%), reflecting stable metabolic turnover. In contrast, the stressed control showed a significant increase (+11.7%), likely due to salt-induced accumulation of stress-related amino acids in the medium, consistent with their roles as osmoprotectants and metabolic regulators under hyperosmotic conditions.

Osmolyte supplementation strongly influences amino acid dynamics. In cultures supplemented with disaccharides (sucrose, trehalose, and lactose) and polyols (mannitol, sorbitol, xylitol, and *myo-*inositol), extracellular amino acid levels consistently decreased after fermentation, reflecting greater uptake and metabolic incorporation. The largest decreases were observed with sorbitol 2%, *myo-*inositol 1%, lactose, xylitol, sucrose 2%, and trehalose 2%, showing reductions of ~28% to 36%. For example, sucrose 2% dropped from 2.64 to 1.83 mg/mL. These decreases correlated with improved growth and viability in the same treatments.

In contrast, direct amino acid supplementation (glycine, proline, and β-alanine) led to poor utilization, with residual concentrations remaining high or even unchanged after 98 h, consistent with their weaker osmoprotective effects. Overall, these results demonstrate that effective osmolytes (disaccharides, polyols, and *myo-*inositol) promoted amino acid uptake and metabolic assimilation, whereas ineffective osmolytes (glycine, proline, β-alanine, and urea) showed little utilization, aligning with their limited impact on stress tolerance.

### Yeast biomass production under salt stress

The effect of osmolyte supplementation on yeast biomass production under 6% NaCl stress was assessed by measuring dry biomass (g/L, DB) after 98 h of fermentation (Fig. S11; Table S3). The unstressed control (−) yielded 2.03 g/L, while the stressed control (+) produced 1.38 g/L, 31.73% reduction due to osmotic stress (Table 2).

**TABLE 2:** Summary of key quantitative metrics of the top three performing osmolytes with controls

Osmolyte (concentration % wt/vol)	Final OD_600_	μmax (h^−^¹)	Doubling time (Td, h)	Viability (CFU × 10⁶)	Budding cells (%)	Cell diameter (µm)	Final biomass (g/L)
*Myo-*inositol (2%)	0.657	0.0494	14.03	23.4	79.90	6.7966	1.98
Sucrose (2%)	0.592	0.0497	13.94	21.9	83.30	7.172	1.73
Lactose (1%)	0.5495	0.0455	15.23	26.4	71.30	6.5228	1.76
Control (+)	0.346	0.0356	19.47	14.54	14.42	4.3056	1.38
Control (−)	1.193	0.1195	5.80	81.21	74.76	7.609	2.03

Osmolyte supplementation at 1% and 2% concentrations showed varied impacts. *Myo-*inositol 2% produced the highest biomass (1.98 g/L, 42.77% increase vs control [+]), followed by proline 1% (1.83 g/L, 32.12%), sucrose 1% and 2% (1.76 g/L, 27.48% and 1.73 g/L; 24.62%). Other notable osmolytes included lactose 2%, glycine 2%, and trehalose 2%. Higher concentrations (2%) improved biomass for *myo-*inositol (27.12%), sucrose (2.29%), lactose (6.70%), trehalose (16.40%), mannitol (4.04%), glycine (47.19%), and glycerol (13.28%), but reduced it for sorbitol (−2.68%), xylitol (−18.51%), proline (−30.77%), and β-alanine (−9.88%). Urea at 0.07% outperformed 0.03% by 16.86%. Overall, the results indicate that supplementation with specific compatible solutes, particularly *myo-*inositol 2%, proline 1%, and sucrose 2%, substantially mitigates the effects of salt stress and supports higher biomass accumulation compared to stressed control.

### Multivariate assessment: PCA

To integrate multivariate responses, PCA was performed using seven standardized variables across 25 treatments: final optical density (OD_600_), CFU per milliliter, dry biomass, reducing sugars, total carbohydrates, radical scavenging activity, and free amino acids. The scree plot showed that PC1 (45.6%) and PC2 (21.1%) together explained 66.7% of the variance, indicating they captured the dominant treatment effects (Fig. S12; Table S4).

**Fig 6:**
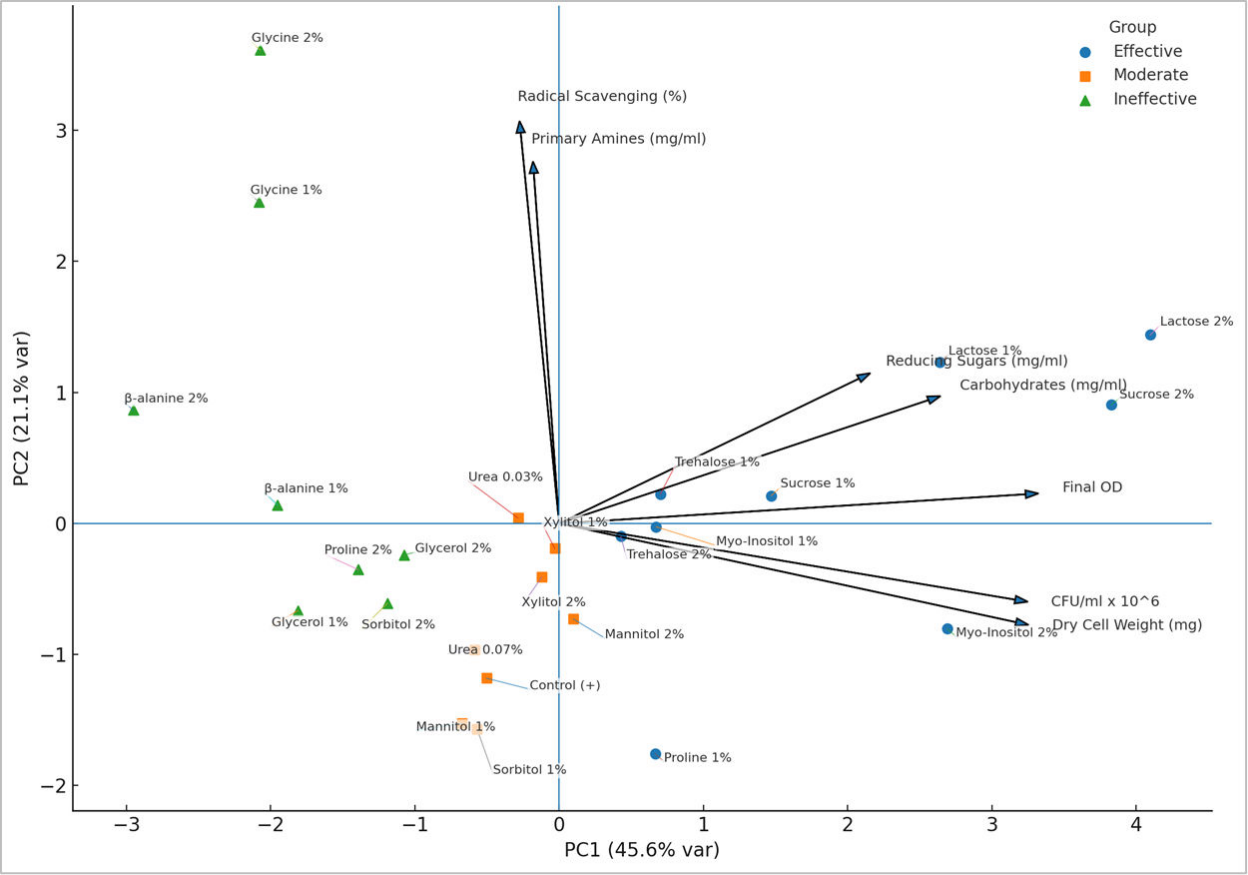
PCA biplot: clustering effective osmolyte treatments and distinct patterns in variable loading directions.

PC1 (growth performance axis) was driven primarily by OD_600_, CFU, and dry biomass, with additional contributions from carbohydrate utilization, suggesting that higher PC1 scores represent treatments that restored growth and viability under salt stress. PC2 (medium chemistry axis) was influenced mainly by initial amino acid content and antioxidant capacity, reflecting biochemical protection rather than growth outcomes. Clear clustering emerged in PCA space (Fig. 6). The effective cluster (top PC1 scorers) included lactose (1%–2%), sucrose (1%–2%), *myo-*inositol (2%), and trehalose (1%–2%), characterized by strong biomass restoration and enhanced carbohydrate turnover. The moderate cluster (middle PC1 values) comprised mannitol (1%–2%), xylitol (1%–2%), urea (0.03%), and sorbitol (1%), which provided partial benefits. The ineffective cluster (bottom PC1 scorers) included β-alanine (1%–2%), glycine (1%–2%), and glycerol (1%), which showed poor growth rescue despite sometimes elevated antioxidant or amino-nitrogen signals. This separation highlights that effective osmolytes combine growth recovery with metabolic balance, while ineffective ones may elevate stress markers without translating into biomass gains.

#### Heatmap corroboration

**Fig 7:**
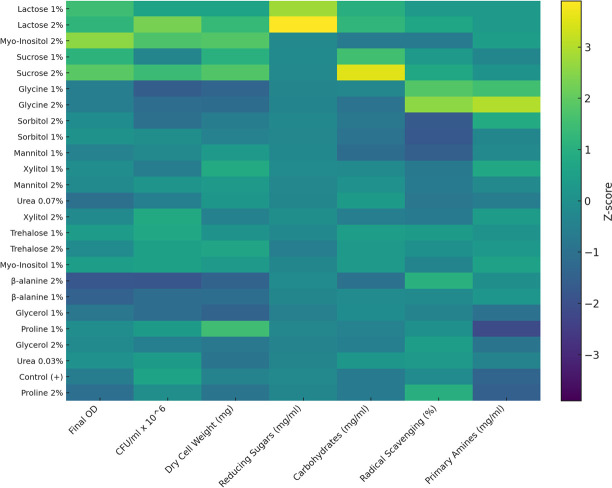
Heatmap of standardized variables for PCA: *S. cerevisiae* under high salt stress.

The row-clustered heatmap of standardized *z*-scores reinforced the PCA-derived groupings (Fig. 7). Effective treatments such as lactose (1%–2%), sucrose (1%–2%), and *myo-*inositol (2%) clustered together with high final OD_600_, CFU, and dry biomass, reflecting strong growth recovery under salt stress. In contrast, ineffective treatments clustered with low biomass indicators despite occasionally elevated primary amines and antioxidant capacity. This pattern demonstrates that biochemical enrichment captured by PC2 does not necessarily translate into growth rescue at 6% NaCl, underscoring the importance of integrated growth metrics when assessing osmolyte efficacy.

## DISCUSSION

The severe impairment of *S. cerevisiae* growth under 6% NaCl reflects the combined hyperosmotic, oxidative, and ionic stresses, leading to water efflux, ion imbalance, and activation of stress response pathways. This is consistent with previous findings that salt stress diverts metabolic resources from proliferation toward survival, reducing growth, viability, and budding (8, 43). Overall, our findings demonstrate that osmolyte efficacy depends on biochemical compatibility with yeast physiology. While the physiological improvements observed are clear, the precise underlying biochemical mechanisms were not directly investigated in this study and may warrant further investigation.

### The efficacy of osmolyte classes and their mechanisms of action

#### Effective osmoprotection: *myo-*inositol and disaccharides (sucrose and lactose)

*Myo-*inositol emerged as the most effective osmolyte, nearly doubling biomass compared to the salt-stressed control. Its efficacy is likely due to its role as a precursor for phospholipid biosynthesis, which supports membrane integrity and phosphoinositide signaling for stress adaptation and mitotic progression (11, 44–46). This is supported by microscopic analysis, where *myo-*inositol treatment restored cell diameter to near unstressed levels. Sucrose and lactose also provided strong protection. Sucrose contributes metabolizable hexoses that can fuel energy-intensive cell division and glycerol biosynthesis, despite partial catabolite repression under stress (47). Lactose acts as a non-metabolized compatible solute, balancing osmotic pressure and stabilizing water structure (48–50). While sucrose is a well-documented metabolizable carbon source for *S. cerevisiae*, lactose is generally considered to be non-metabolizable by most common strains of this species. Our results show that both disaccharides provide significant protection against salt stress. This suggests that the benefits of lactose in this study might stem primarily from its role as a physical compatible solute. Such solutes are hypothesized to stabilize cellular membranes and proteins through the water replacement or preferential exclusion models, providing protection even if they are not utilized as a primary energy source (23, 51). Both disaccharides restored budding rates to nearly double that of the stressed control, suggesting improved cellular homeostasis.

### Moderate osmoprotection: trehalose and polyols

Trehalose provided moderate benefits, primarily through its role as a chemical chaperone that stabilizes proteins and membranes (52–55). However, benefits plateaued at higher concentrations, possibly due to limited uptake or inhibition of its synthesis pathway (56). Polyols like sorbitol and mannitol showed modest effects, acting as non-native compatible solutes with limited intracellular metabolic roles (57). Interestingly, xylitol showed stronger effects than sorbitol and mannitol, suggesting a secondary role in redox balancing in NAD(*P*)H metabolism (58).

### Limited osmoprotection: amino acids and urea

Amino acids presented divergent outcomes. Under high-glucose osmotic stress (35% wt/vol), glycine restored viability to ≥80% and sugar utilization to 92%, outperforming proline (27% viability) (59), aligning with our PCA clustering. Proline and glycine offered partial protection, consistent with their roles in oxidative stress buffering and protein stabilization (27, 60). In line with reports on ethanol-induced osmotic stress, intracellular L-proline accumulation restored yeast viability to ~75% (61), supporting our findings where proline 1% gave higher dry cell weight compared to other amino acids. However, higher concentrations could be inhibitory, highlighting the risk of nitrogen imbalance (62). β-Alanine was largely ineffective, reflecting poor metabolic integration (63). High levels of urea were also inhibitory, possibly due to ammonia release disrupting pH homeostasis (64). The ineffectiveness of exogenous glycerol, despite being the native osmolyte, is consistent with the requirement for endogenous synthesis for osmoprotection, as its uptake is restricted by channel regulation under stress (65, 66).

### Biophysiological correlates of osmoprotection

#### Budding and cell morphology

The drastic (~80%) reduction in budding observed in the stressed control highlights the inhibitory effect of osmotic stress on cell cycle progression, consistent with earlier findings (8, 11). The effective osmolytes, particularly disaccharides (sucrose and lactose) and *myo-*inositol, provided the most robust protection, restoring budding cell percentages to nearly double that of the stressed control, suggesting improved cellular homeostasis and metabolic activity. This is consistent with morphological analysis, where salt-stressed cells showed pronounced shrinkage and plasmolysis, and *myo-*inositol, sucrose, and lactose were able to restore cell diameters to near unstressed levels.

### Sugar and carbohydrate utilization

Analysis of sugar consumption revealed that hyperosmotic stress suppresses glycolytic activity, with the stressed control consuming only ~34% of available sugars compared to 97% in the unstressed control. Effective osmolytes like *myo-*inositol, trehalose, and lactose substantially improved sugar consumption efficiency, bringing utilization rates closer to the unstressed control. *Myo-*inositol enabled 83.7% sugar consumption, a 2.5-fold improvement over the stressed control. This finding suggests that effective osmoprotection enables a form of metabolic rescue where the cell can maintain crucial nutrient uptake and energy production pathways. The unique behavior of sucrose at higher concentrations, which resulted in a net sugar accumulation, is consistent with extracellular invertase activity that may exceed uptake capacity (67).

### Oxidative stress and antioxidant defenses

Oxidative stress is a major consequence of high-salt environments in yeast, leading to the depletion of antioxidant defenses (68). Osmolyte supplementation had distinct effects on the %RSA of *S. cerevisiae* under salt stress, highlighting differences in how compounds contribute to redox balance. The stressed control exhibited a significant decline in its ability to scavenge free radicals. As expected, the unstressed control displayed an increase in antioxidant capacity over time, reflecting the natural induction of antioxidant defenses during fermentation. In contrast, the stressed control exhibited a significant decline (−18.6%), consistent with NaCl-induced ROS accumulation that oxidizes lipids, proteins, and nucleic acids (8). Among treatments, glycine provided the strongest antioxidant response, enhancing RSA by 47% and resulting in higher biomass (1.65 g/L). This effect likely reflects its dual role as a precursor for glutathione, a key antioxidant, and as a direct scavenger of free radicals (27). Despite this, glycine offered only minimal growth benefits. This indicates that an osmolyte’s ability to mitigate oxidative stress does not in itself guarantee improved proliferation and that salt stress is a multifaceted challenge.

Disaccharides contributed through combined osmotic and redox mechanisms. Sucrose maintained RSA (+9.6%), likely by reducing stress on membrane-bound enzymes via extracellular buffering rather than direct ROS quenching (47). Lactose produced similar effects (+7%), functioning as a compatible solute without metabolic interference. Proline showed modest benefits, consistent with its known role in stabilizing proteins and scavenging hydroxyl radicals (27). Unexpectedly, trehalose underperformed (−7% to −10%), potentially due to inhibition of trehalose-6P synthase (Tps1) under salt stress, limiting its ability to accumulate and protect cells (56). Glycerol supplementation was similarly ineffective (−9.2% at 2%), reflecting HOG pathway saturation and restricted glycerol transport during osmotic stress (65). The most effective osmolytes like *myo-*inositol, sucrose, and lactose appear to provide a more holistic form of cellular protection, addressing multiple facets of the stress response simultaneously, leading to restored growth and productivity.

### Multivariate analysis confirms integrated responses

The PCA provides a statistically robust confirmation of our findings. The multivariate PCA revealed that yeast adaptation to high salt stress can be described along two orthogonal dimensions, the separation of treatments along PC1, which accounts for 45.6% of the variance and is driven by growth-related variables (OD_600_, CFU, and biomass), validates that growth performance is the dominant factor separating the treatments, reflecting the capacity of osmolytes to restore proliferation under osmotic stress. PC2 (medium chemistry) captured variation in baseline amino acids and antioxidant capacity. The separation of these axes underscores that biochemical enrichment (e.g., high amino acids or antioxidants) does not by itself restore growth unless it directly supports osmoadaptation.

The clustering of *myo-*inositol, sucrose, and lactose with the unstressed control on the PCA biplot is a strong statistical statement about their superior osmoprotective potential. Conversely, ineffective treatments like β-alanine, glycine, and glycerol form a distinct cluster. This analysis elegantly summarizes that compounds that promote growth share a common metabolic and physical profile, whereas those that do not fail across a range of metrics. These findings indicate that *S. cerevisiae* preferentially benefits from disaccharides and polyols rather than amino acids for osmoprotection, contrasting with the strategies of many bacteria and plants (27, 69).

Beyond direct osmoprotection, the modulation of fermentation conditions significantly impacts downstream metabolite profiles. Recent studies highlight the broader implications of microbial osmotic stress tolerance in industrial fermentation, where strategies such as physical immobilization or cocultivation can enhance yield. In pineapple wine production, sequential inoculation with non-*Saccharomyces* yeast under high-sugar conditions significantly increased glycerol production (~1.26 g/L) compared to *Saccharomyces cerevisiae* alone, improving overall product quality and suggesting an intrinsic metabolic shift toward osmolyte accumulation (70). Similarly, enhancement of yeast stress tolerance through immobilization on nutrient-rich matrices increased bioethanol production by over 21%, effectively reducing the lag phase associated with environmental stress (71). Our findings align with this trend, showing that exogenous osmolyte supplementation acts as a parallel strategy to preserve cell viability and sustain biomass accumulation under high-osmolarity conditions.

In *S. cerevisiae*, adaptation to osmotic stress involves an intricate interplay between ion transport, vacuolar acidification, and redox homeostasis. The proton-translocating vacuolar-ATPase (V-ATPase) and the plasma-membrane ATPase (Pma1) are central to maintaining intracellular pH and ionic equilibrium during high-salt exposure. Previous studies have shown that V-ATPase activity operates in concert with the HOG pathway to restore cellular turgor and cytoplasmic pH following osmotic shock (72). Disruption of this system or altered Na^+^/K^+^ flux regulation can compromise vacuolar integrity and overall stress tolerance (73, 74). The accumulation of compatible solutes observed in this study likely complements these homeostatic processes by stabilizing macromolecular structures and indirectly supporting ATPase-driven proton pumping and ion balancing (75, 76). Although the present work focused on comparative physiological responses rather than direct biochemical assays, our findings are consistent with the established model in which osmolyte-mediated growth recovery reflects enhanced energy metabolism and proton-gradient maintenance across membranes (77, 78).

During osmotic stress, *Saccharomyces cerevisiae* activates the HOG pathway, inducing glycerol biosynthesis to restore turgor pressure (8, 79). This adaptive response requires substantial metabolic investment, as glycerol synthesis diverts carbon away from glycolysis and consumes reducing equivalents (NADH), thereby increasing energetic demand during stress conditions (77). By utilizing exogenous compatible solutes via active transporters like Stl1p, the availability of osmolytes can partially alleviate this metabolic burden by reducing the need for *de novo* glycerol production, allowing cells to maintain osmotic balance while reallocating metabolic resources toward growth and cellular maintenance (14, 80–82). Future studies integrating direct measurements of ATPase activity and ion fluxes will further elucidate how individual osmolytes interface with these conserved osmoadaptive mechanisms.

### Conclusion

This study provides the first direct comparative ranking of commercial osmolytes for mitigating high-salt stress in *S. cerevisiae*. Among the twelve osmolytes tested, disaccharides (sucrose, lactose, and trehalose) and *myo-*inositol emerged as the most effective osmoprotectants, restoring growth, viability, and metabolic activity to levels approaching unstressed controls. These osmolytes enhanced budding frequency, preserved cell morphology, and improved sugar utilization while simultaneously reinforcing antioxidant defenses. Osmolytes that integrate into central metabolism (sucrose and *myo-*inositol) or stabilize macromolecules (trehalose and lactose) provided the greatest benefits, while amino acids and urea were largely ineffective.

Multivariate analyses confirmed these trends, clustering disaccharides and *myo-*inositol with unstressed cells, while ineffective osmolytes grouped with salt-stressed controls. Collectively, these findings establish that targeted osmolyte supplementation, particularly with *myo-*inositol, sucrose, and lactose, represents a practical and scalable strategy to mitigate osmotic stress. From an applied perspective, such supplementation provides a quantitative framework for selecting osmoprotectants to improve industrial productivity while also considering factors such as concentration and cost for large-scale implementation in saline or high-osmolarity fermentations, like high-gravity brewing, bioethanol from molasses or DDGS, fruit processing residues, whey permeate, and dairy byproducts.

Future work should explore combinatorial supplementation strategies, where synergistic osmolytes may offer superior protection compared to single compounds. Future work should also investigate ion fluxes, V-ATPase activity, and intracellular osmolyte accumulation to elucidate the precise mechanisms underlying the superior performance of disaccharides and *myo-*inositol. Expanding studies to bioreactor-scale fermentations will help validate scalability and process efficiency under dynamic industrial conditions. Finally, investigating osmolyte performance in other industrially important microorganisms will broaden the applicability of these findings to diverse biotechnological platforms.

## Data Availability

Data will be made available on request.

## SUPPLEMENTAL MATERIAL

Supplemental figures and tables spectrum.00102-26-s0001.pdf Figures S1 to S13 and Tables S1 to S4.

Graphical abstract spectrum.00102-26-s0002.docx Graphical abstract.

ASM does not own the copyrights to Supplemental Material that may be linked to, or accessed through, an article. The authors have granted ASM a non-exclusive, world-wide license to publish the Supplemental Material files. Please contact the corresponding author directly for reuse.
