# Supplementary material for: Comparative assessment of food-grade osmolytes for enhancing yeast fermentation performance under salt stress
Source: Microbiol Spectr. 2026 Mar 30;14(5):e00102-26. doi: 10.1128/spectrum.00102-26 (PMC13141918; doi:10.1128/spectrum.00102-26)
Supplement: Supplemental figures and tables — Figures S1 to S13 and Tables S1 to S4. [file spectrum.00102-26-s0001.pdf]

SUPPLEMENTARY INFORMATION

Comparative Assessment of Food Grade Osmolytes for Enhancing Yeast Fermentation Performance Under Salt Stress

G. Sathaiah[1^], P. Chen[1^], M. Khant[1], P. Pillai[1*]

Supplementary Figures:

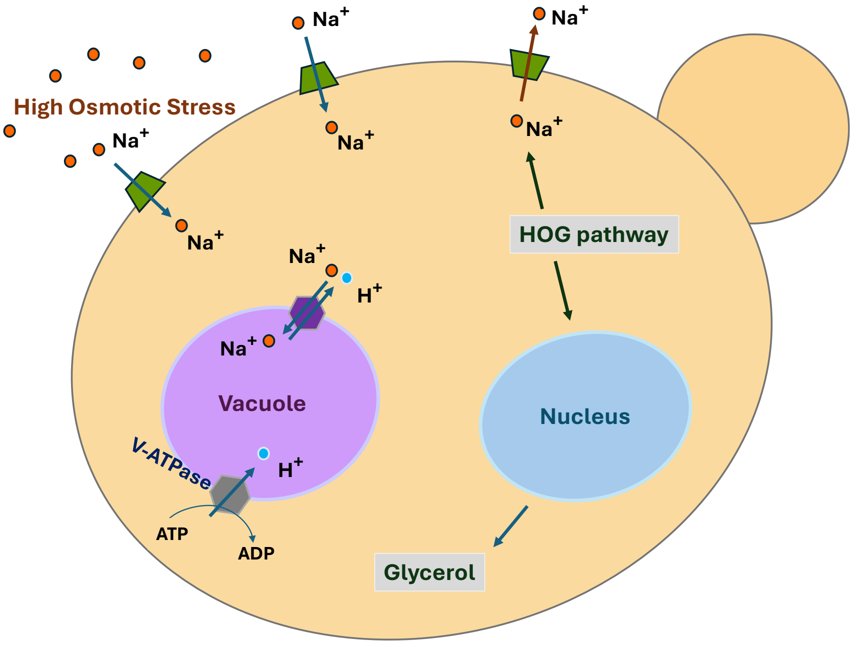

Figure S1. Mechanistic Model of V-ATPase Activation and HOG Pathway Response in *Saccharomyces cerevisiae* under Osmotic Stress, adapted and modified from Li et al. (2012) [15].

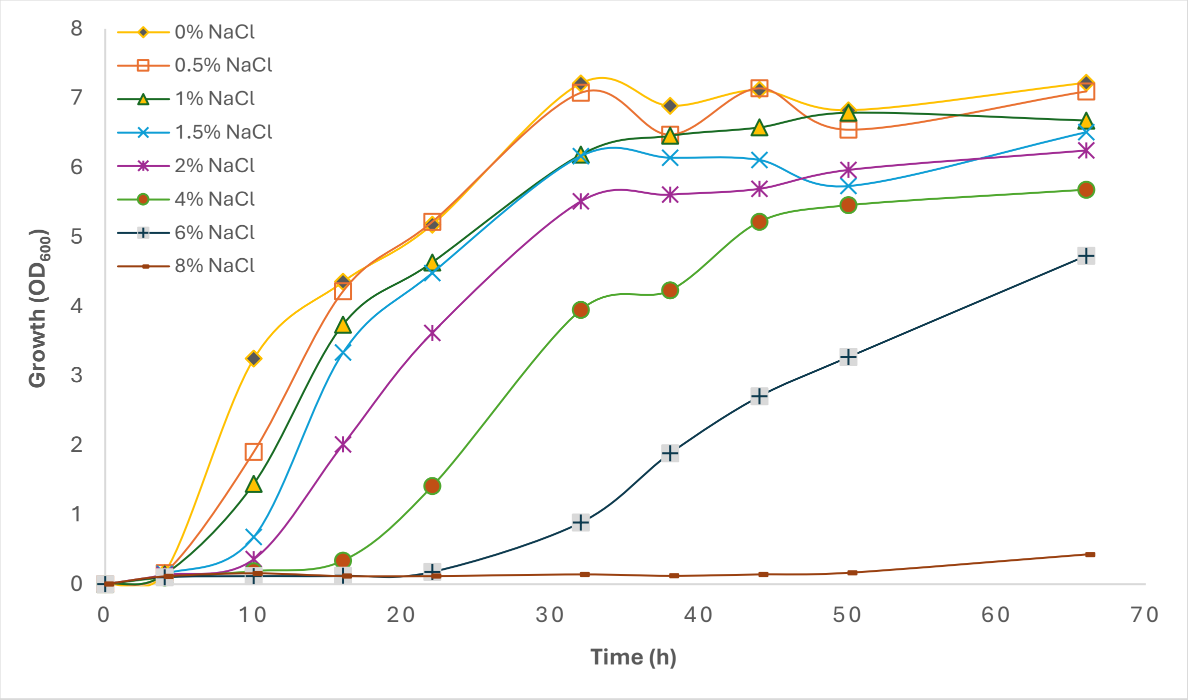

Figure S2. Yeast growth kinetics at different salt (NaCl) concentrations in YPD medium.

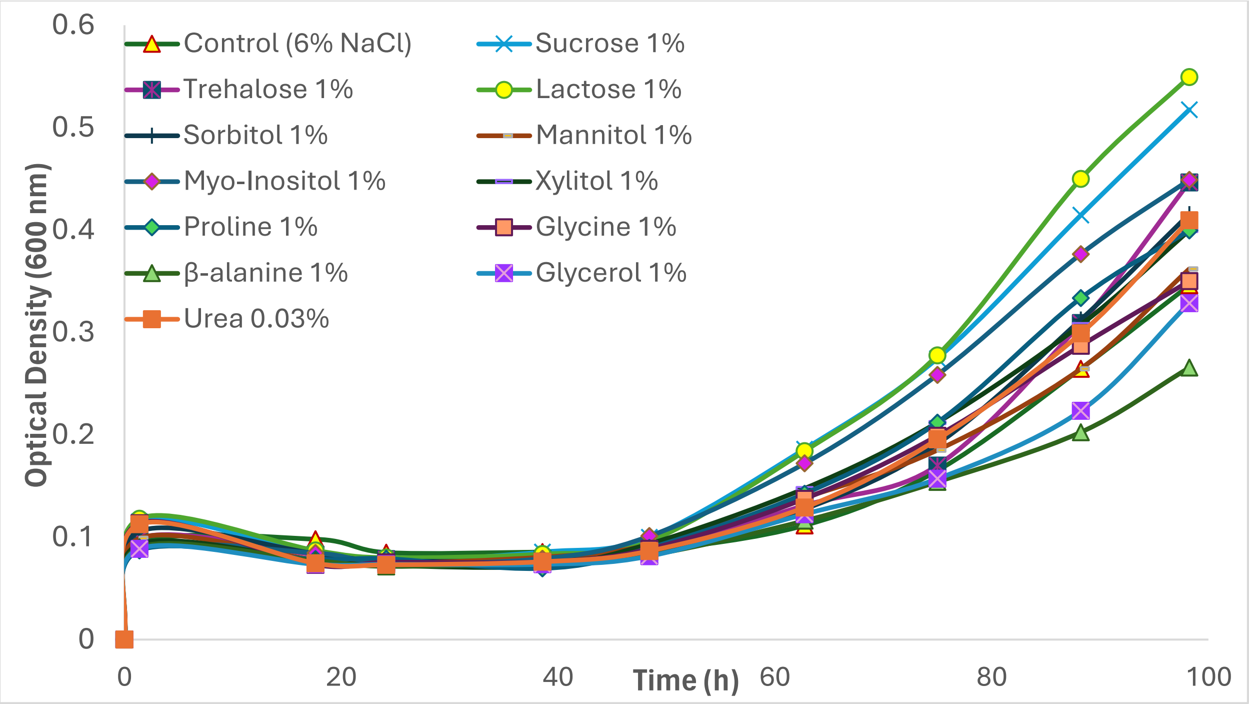

Figure S3. Comparison of growth kinetics for the osmolytes (1%) in *S. cerevisiae* under high salt stress.

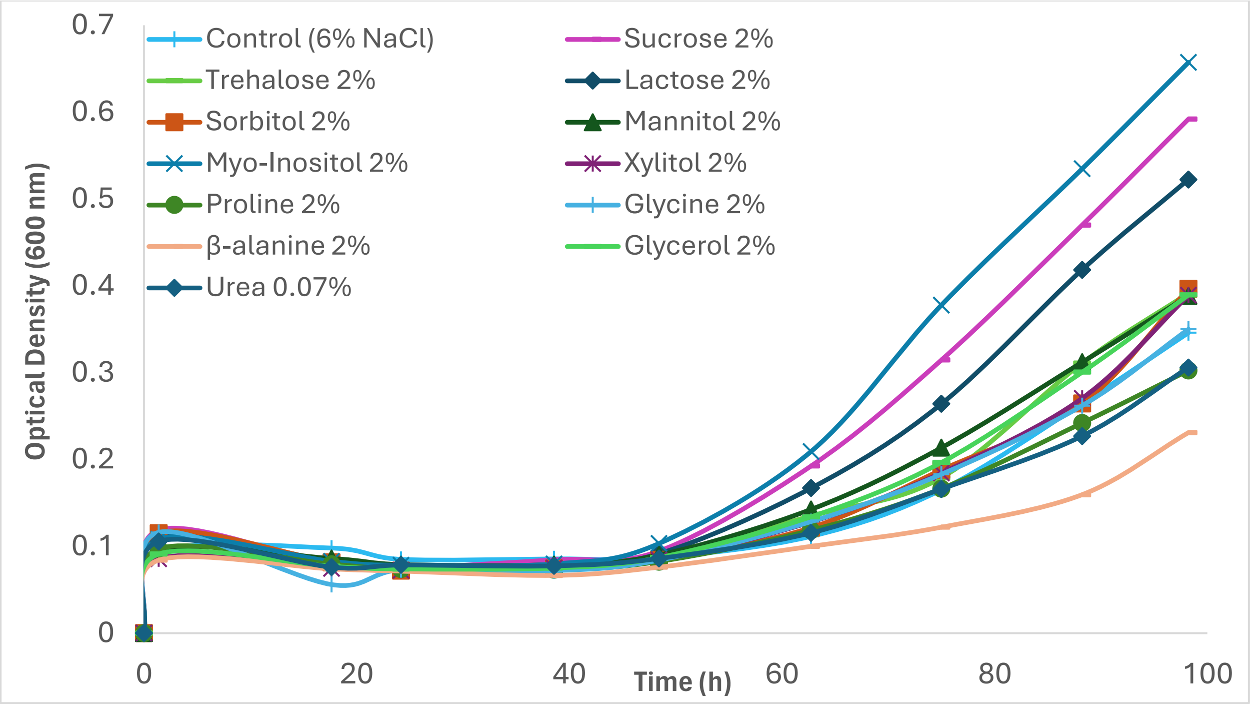

Figure S4. Comparison of growth kinetics for the osmolytes (2%) in *S. cerevisiae* under high salt stress.

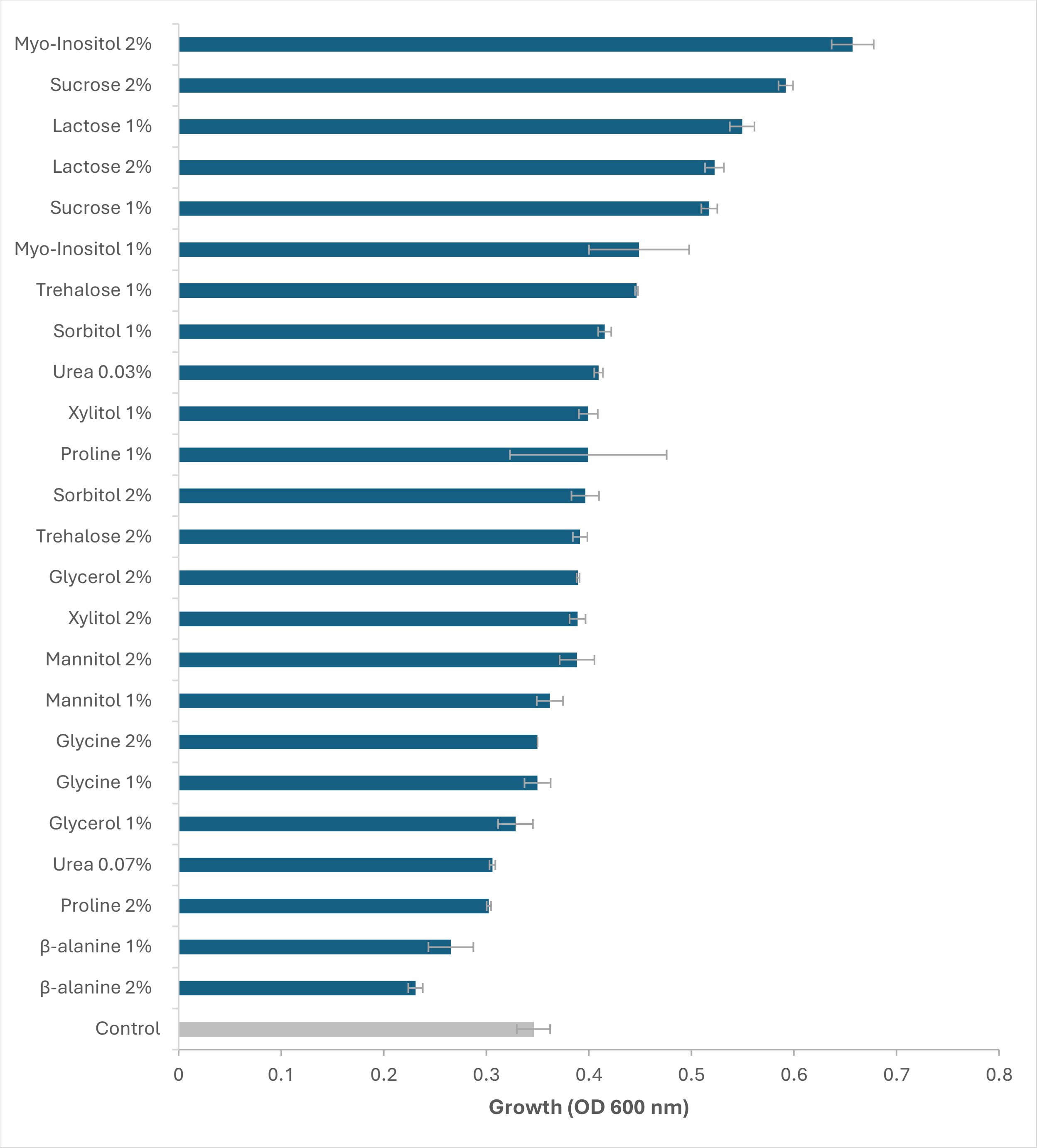

Figure S5. Comparative final growth (OD_{600}) of *S. cerevisiae* under 6% NaCl with twelve different osmolytes at 1% and 2% (w/v).

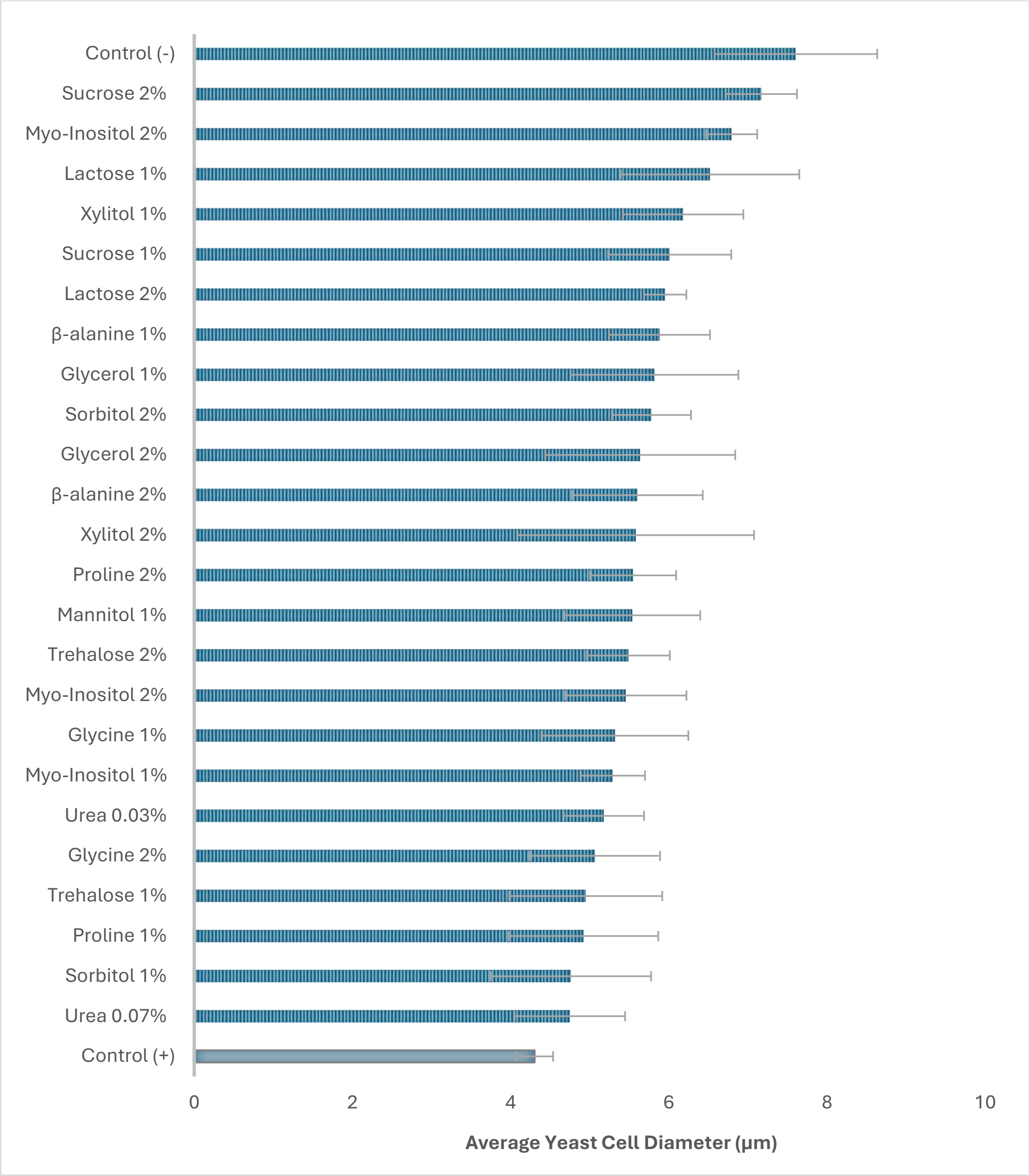

Figure S6. Yeast cell diameter profile under salt stress with osmolyte supplementation. (n=5)

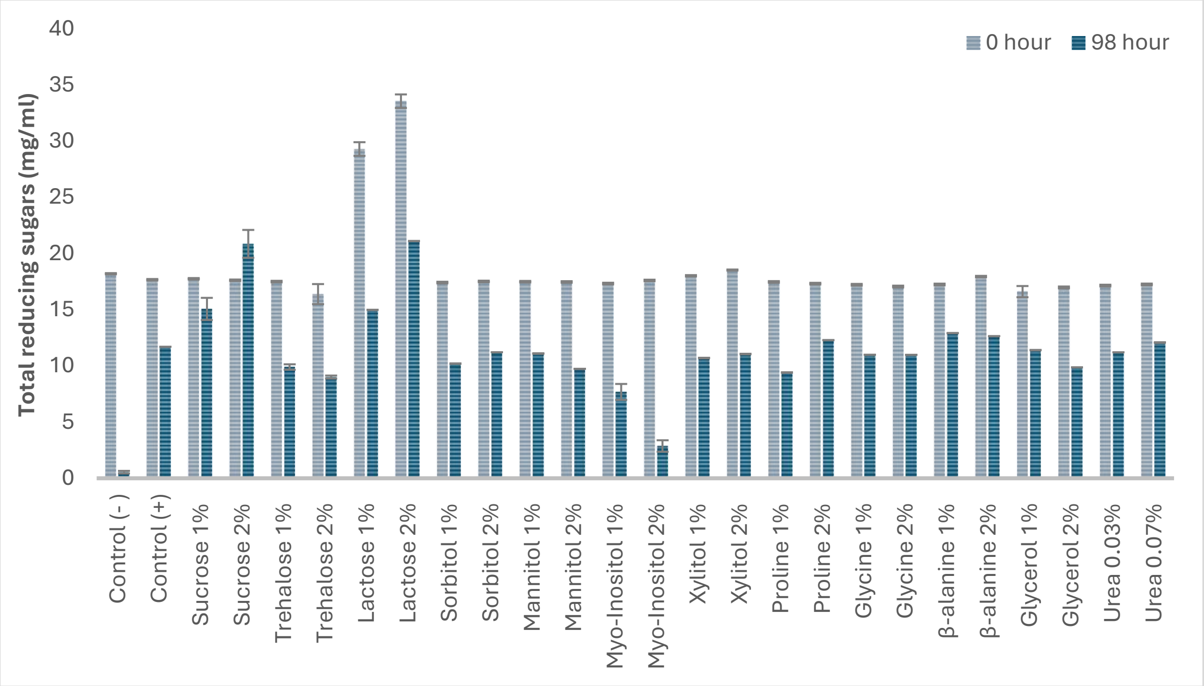

Figure S7. Effect of Osmolytes on Reducing Sugar Dynamics in *S. cerevisiae* under Salt Stress (0 h vs 98 h).

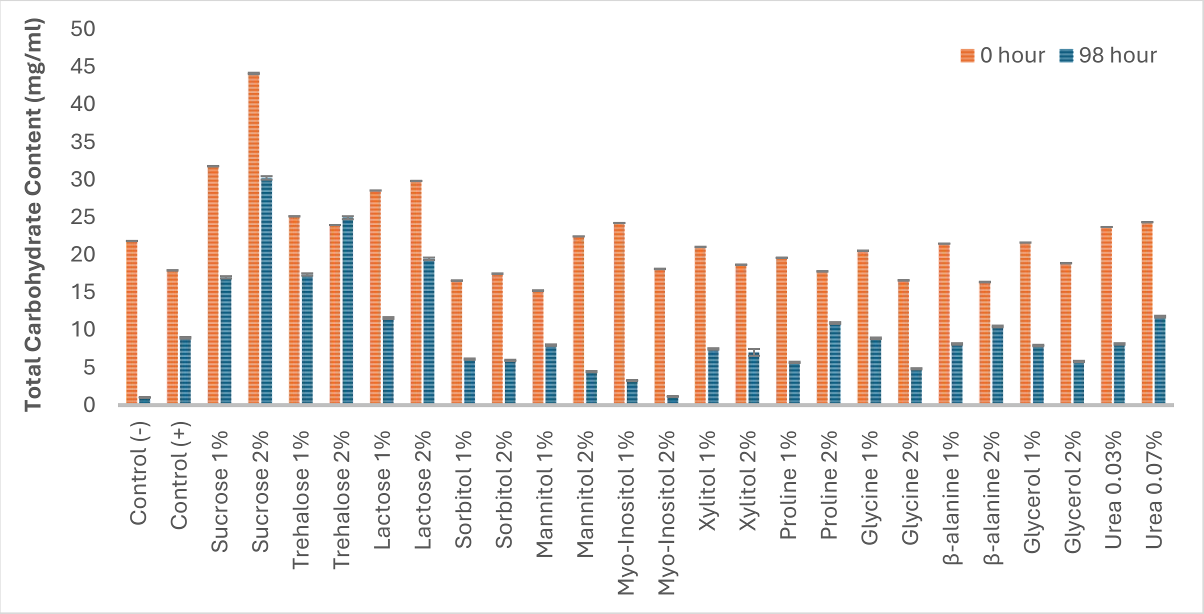

Figure S8. Total carbohydrates profiles before and after fermentation across osmolyte conditions.

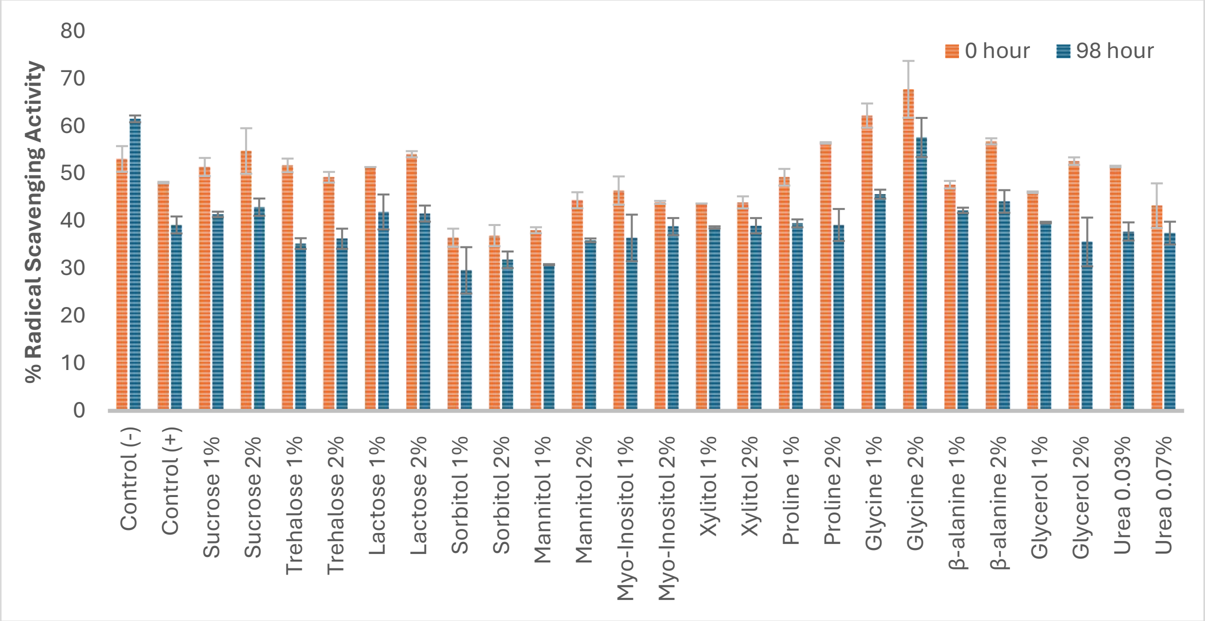

Figure S9. Effect of Osmolytes on Radical Scavenging Activity under Salt Stress (0 vs 98 h).

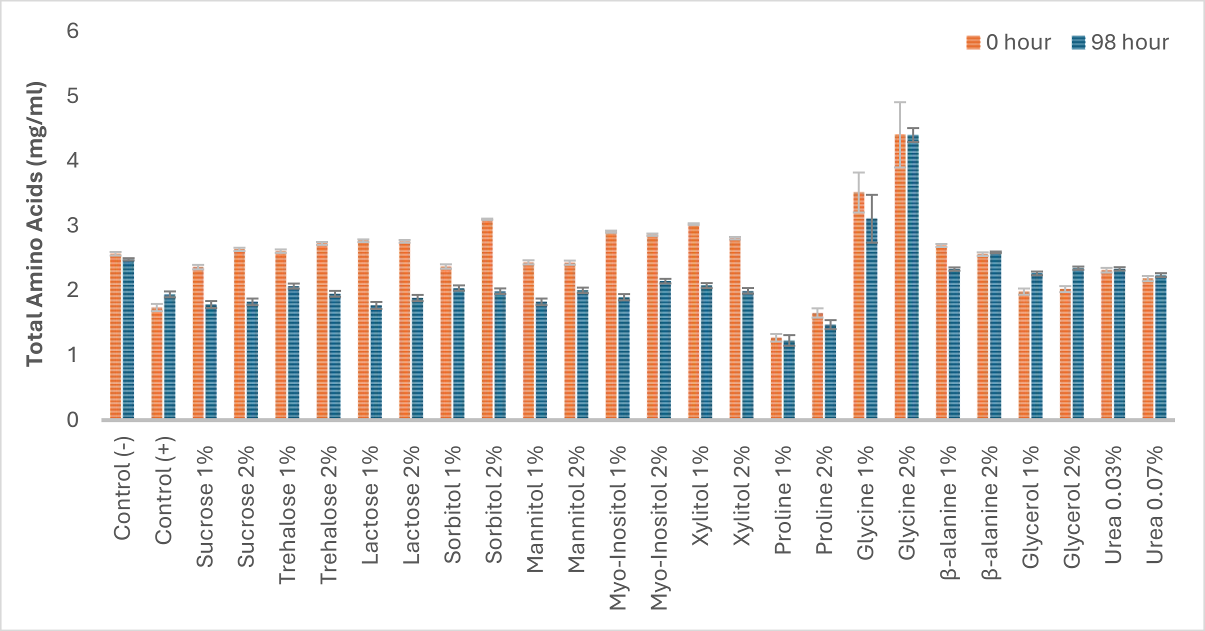

Figure S10. Effect of Osmolytes on free amino acids content under Salt Stress (0 h vs 98 h).

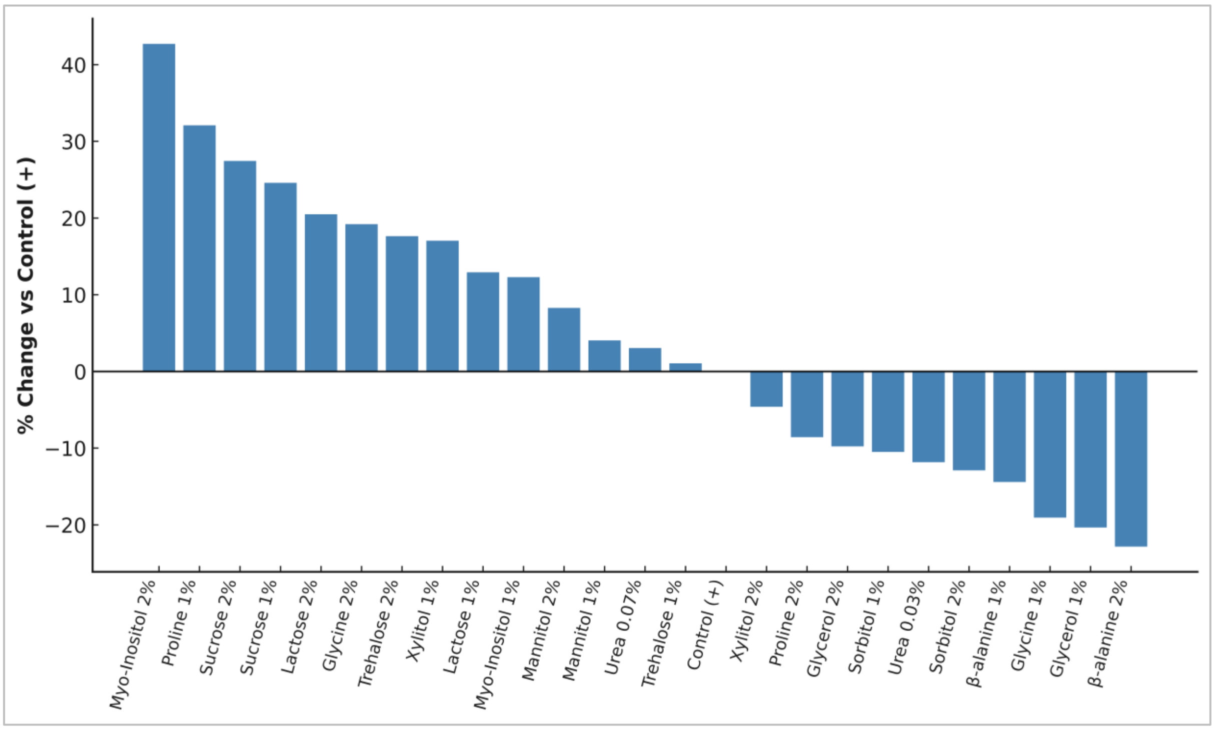

Figure S11. Ranked bar chart: Percentage change in yeast biomass compared to Control (+).

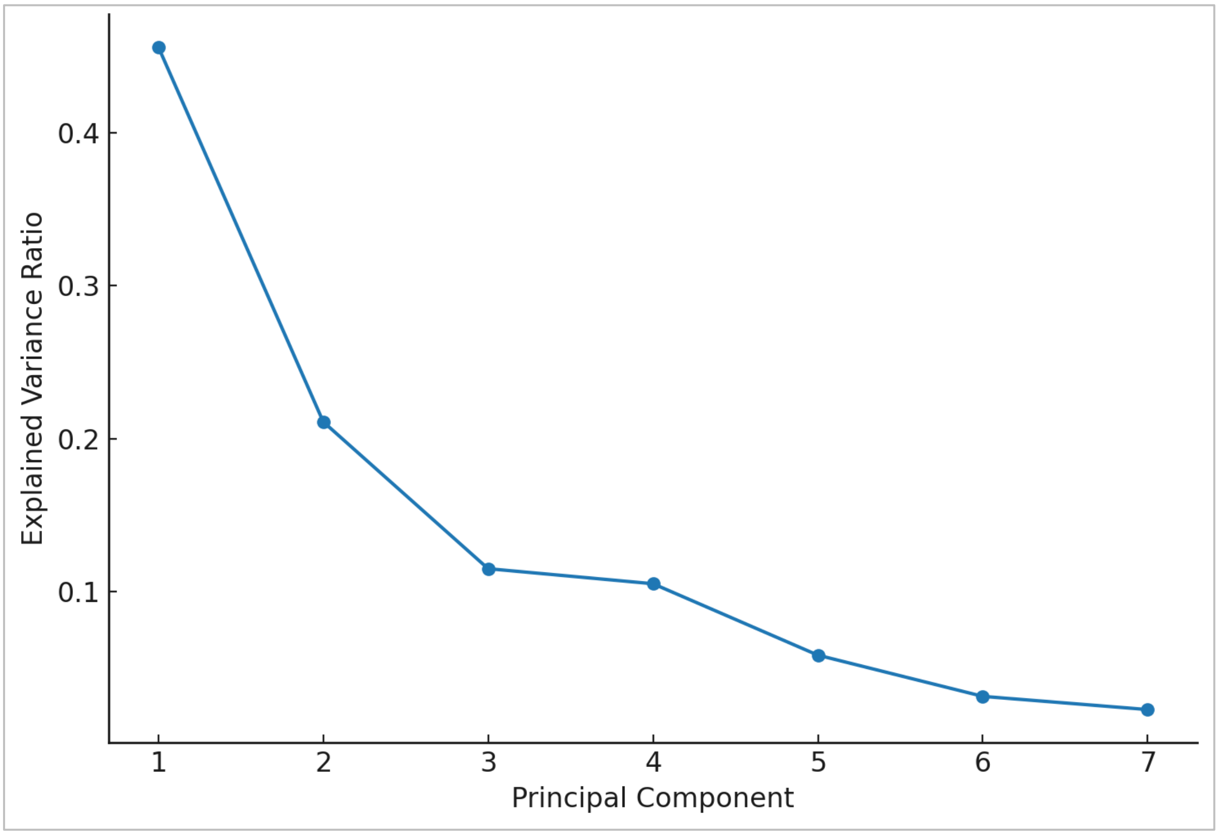

Figure S12. PCA: Scree Plot Analysis

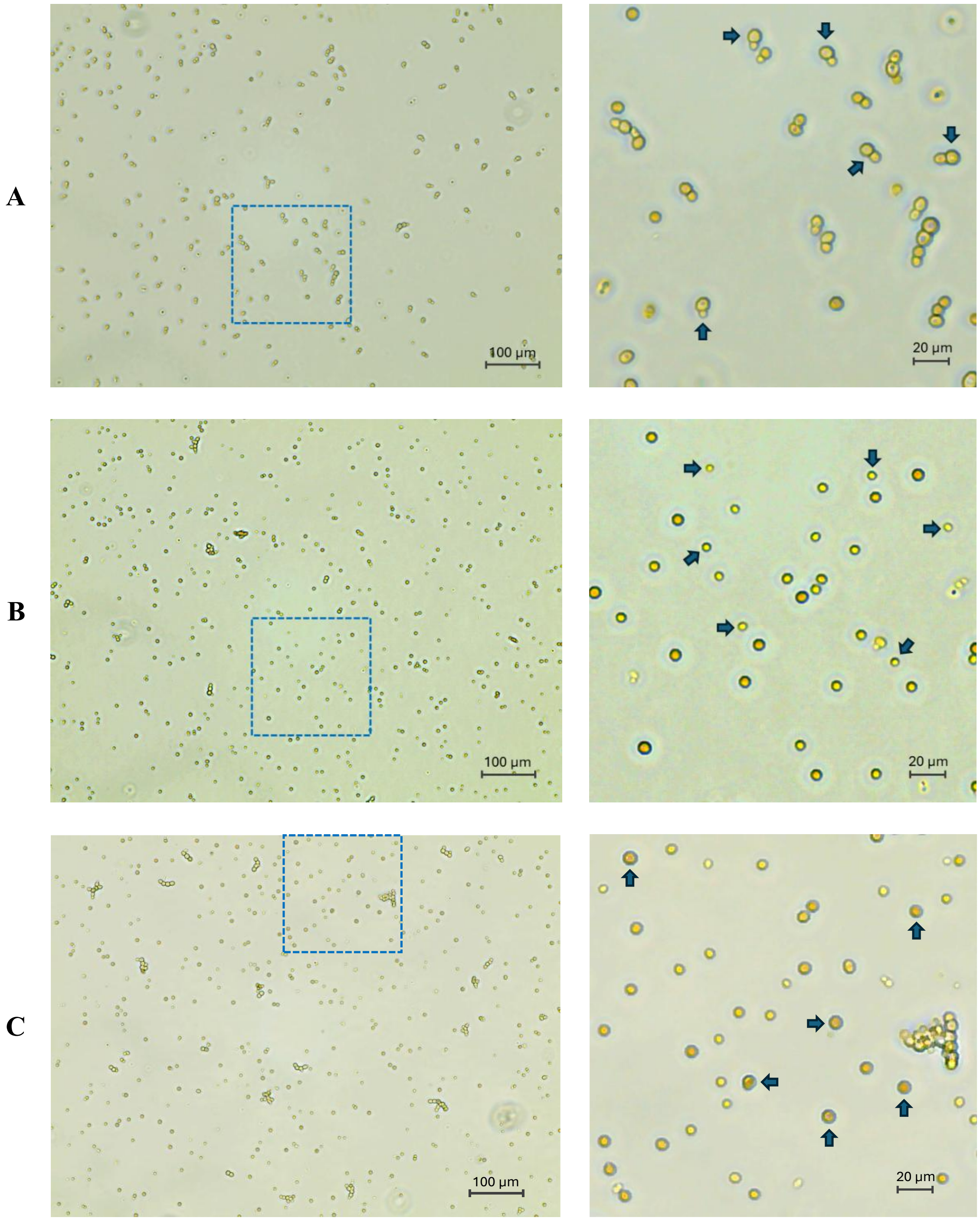
A
100 µm
20 µm
B
100 µm
20 µm
C
100 µm
20 µm

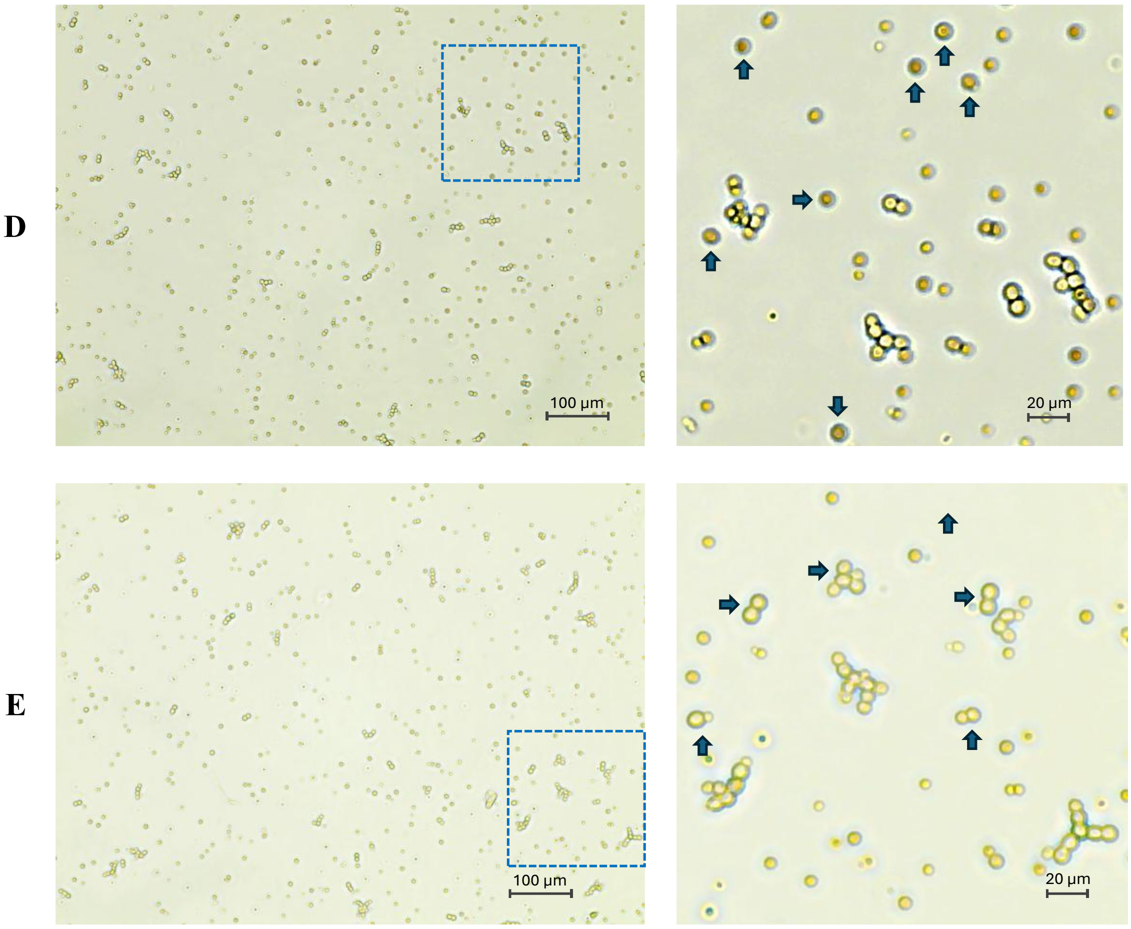

Figure S13. Bright-field microscopy images (200×) showing cell morphology of *S. cerevisiae* under different osmotic conditions. ***A.*** *Unstressed control (0% NaCl);* ***B.*** *NaCl-stressed control (6% NaCl);* ***C.*** *Myo-inositol (2%);* ***D.*** *Sucrose (2%);* ***E.*** *Lactose (1%). Blue arrows indicate enlarged cells reflecting recovery under osmolyte supplementation.*

Supplementary Tables:

Table S1. Comparative efficacy of osmolytes (at 1 and 2% w/v) on *S. cerevisiae* growth

Osmolyte (2%)	Final OD_{600} (98 h)	Specific Growth Rate (μ) (h^{-1})	Improvement in Growth Rate (%)
**Myo-Inositol**	0.657	0.0413	85.20%
**Sucrose**	0.592	0.0401	79.80%
**Lactose**	0.523	0.0381	70.90%
**Trehalose**	0.447	0.0314	40.80%
**Sorbitol**	0.396	0.0287	28.70%
**Proline**	0.303	0.0271	21.50%
**Urea (0.07%)**	0.306	0.0246	10.30%
**Control (+)**	0.346	0.0223	-

Osmolyte (1 %)	Final OD_{600} (98 h)	Specific Growth Rate (μ) (h^{-1})	Improvement in Growth Rate (%)
**Lactose**	0.55	0.0388	74.00%
**Sucrose**	0.518	0.0357	60.10%
**Myo-Inositol**	0.449	0.0331	48.40%
**Proline**	0.4	0.033	48.00%
**Sorbitol**	0.416	0.0324	45.30%
**Trehalose**	0.447	0.0323	44.80%
**Urea (0.07%)**	0.334	0.03	34.50%
**Xylitol**	0.4	0.0299	34.10%

**Glycine**	0.35	0.0296	32.70%
**Mannitol**	0.365	0.0264	18.40%
**Glycerol**	0.329	0.0253	13.50%
**β-alanine**	0.266	0.0226	1.30%
**Control (+)**	0.346	0.0223	-

Table S2. Cell viability CFU/ml (× 10^6) and % Viability relative to Control (-) at 108 h

Rank	Osmolyte Class	Sample	CFU/ml (× 10^6)	% Viability vs. C0
-	Controls	C(-) (0% NaCl)	81.21	100.00%
1	Disaccharides	Lactose 2%	21.41	26.40%
2	Polyols	Myo-Inositol 2%	18.99	23.40%
3	Disaccharides	Sucrose 2%	17.78	21.90%
4	Polyols	Xylitol 2%	15.35	18.90%
5	Disaccharides	Trehalose 1%	14.95	18.40%
6	Disaccharides	Lactose 1%	14.54	17.90%
-	Controls	C(+) (6% NaCl)	14.54	17.90%
7	Disaccharides	Trehalose 2%	14.14	17.40%
8	Polyols	Myo-Inositol 1%	14.14	17.40%
9	Amino Acids & Urea	Proline 1%	13.33	16.40%

10	Amino Acids & Urea	Urea 0.03%	13.33	16.40%
11	Polyols	Mannitol 2%	12.52	15.40%
12	Polyols	Sorbitol 1%	11.72	14.40%
13	Amino Acids & Urea	Proline 2%	11.72	14.40%
14	Polyols	Mannitol 1%	10.91	13.40%
15	Disaccharides	Sucrose 1%	10.5	12.90%
16	Amino Acids & Urea	Glycine 2%	9.7	11.90%
17	Amino Acids & Urea	Urea 0.07%	9.7	11.90%
18	Polyols	Xylitol 1%	9.29	11.40%
19	Polyols	Sorbitol 2%	7.68	9.50%
20	Amino Acids & Urea	β-Alanine 1%	7.27	9.00%
21	Amino Acids & Urea	Glycine 1%	7.27	9.00%
22	Polyols	Glycerol 2%	7.27	9.00%
23	Polyols	Glycerol 1%	5.25	6.50%
24	Amino Acids & Urea	β-Alanine 2%	4.44	5.50%

Table S3. Yeast Biomass Production NaCl Stress with Osmolyte Supplementation

Sample	Yeast Biomass (g/L, DB)	% Change vs. Control (+)
**Control (+)**	1.3852	-
**Control (-)**	2.0286	46.45
**Myo-Inositol 2%**	1.9769	42.77
**Proline 1%**	1.8296	32.12
**Sucrose 2%**	1.7655	27.48
**Sucrose 1%**	1.7259	24.62
**Lactose 2%**	1.6690	20.52
**Glycine 2%**	1.6510	19.18
**Trehalose 2%**	1.6296	17.67
**Xylitol 1%**	1.6214	17.07
**Lactose 1%**	1.5643	12.94
**Myo-Inositol 1%**	1.5556	12.30
**Mannitol 2%**	1.5000	8.29
**Mannitol 1%**	1.4414	4.06
**Urea 0.07%**	1.4276	3.06
**Trehalose 1%**	1.4000	1.07
**Xylitol 2%**	1.3214	-4.59
**Glycerol 2%**	1.2500	-9.74
**Sorbitol 1%**	1.2400	-10.46
**Urea 0.03%**	1.2214	-11.81
**Sorbitol 2%**	1.2067	-12.88

**β-Alanine 1%**	1.1857	-14.38
**Glycine 1%**	1.1214	-19.03
**Glycerol 1%**	1.1036	-20.32
**β-Alanine 2%**	1.0690	-22.83

Table S4. PCA: Variance Explained

Principal Component	Variance Explained (%)	Cumulative Variance (%)
**PC1**	45.62	45.62
**PC2**	21.10	66.73
**PC3**	11.50	78.22
**PC4**	10.51	88.73
**PC5**	5.84	94.57
**PC6**	3.15	97.72
**PC7**	2.28	100.00

The majority of information is captured by the first two principal components (PC1+PC2 = 66.73%).
