# Supplementary material for: Comparative assessment of food-grade osmolytes for enhancing yeast fermentation performance under salt stress
Source: Microbiol Spectr. 2026 Mar 30;14(5):e00102-26. doi: 10.1128/spectrum.00102-26 (PMC13141918; doi:10.1128/spectrum.00102-26)
Supplement: Graphical abstract [file spectrum.00102-26-s0002.docx]

Yeast response to high salt stress and the protective effects of osmolytes.
